# Dynamics of collective creativity in AI art competitions

**DOI:** 10.1093/pnasnexus/pgag369

**Published:** 2026-10-23

**Authors:** Mason Youngblood, Jeff Nusz, Joel Simon

**Affiliations:** Institute for Advanced Computational Science, Stony Brook University, Stony Brook, NY, USA; Morphogen, Berkeley, CA, USA; Morphogen, Berkeley, CA, USA

**Keywords:** cultural evolution, iterated learning, creativity, generative AI, collective behavior

## Abstract

Creativity is a fundamental part of cultural evolution, but the mechanisms by which groups produce novelty are notoriously difficult to infer from the historical record. Iterated learning experiments have shown that cultural transmission reliably distorts artifacts toward the inductive biases of learners, but most of this work uses linear chains of human participants, and it is unclear how these dynamics play out in the networked, human–AI systems that increasingly shape cultural production. In this study, we analyze one such system: Artbreeder, which hosts daily “remix parties” in which users iteratively build on each other’s work from a single seed image, producing branching lineages of images co-created by humans and generative AI. Using 130,882 images from 368 remix parties over 13 months, we find that images become less novel over the course of a party and that each remix step moves less far from its parents, consistent with the stabilization seen in iterated learning experiments, and that late-stage images drift toward the platform’s dominant portrait style. More novel “parent” images produce more novel and more complex “children.” More novel children attract more likes but are not remixed more, whereas more complex children are remixed less. Finally, larger remix parties produce images that are more novel but less complex.

Significance Statement Creativity is fundamental to cultural evolution, but how groups produce novelty is difficult to infer from the historical record. Platforms where users build on one another’s work with generative AI are natural examples of networked, human–AI transmission. We analyze 130,882 images from 368 remix parties on Artbreeder, measuring novelty and complexity with density estimation and segmentation, and relating each image’s properties to its parents and its success. Over a party, images become less novel and each remix moves less far from its parents, consistent with iterated learning studies. More novel images attract more likes but are not remixed more, and simpler images are what users build on. Larger populations produce more novelty at the cost of complexity.

## Introduction

Creativity is a fundamental part of cultural evolution because it generates the variation that other processes select for or against. The mechanisms by which groups produce novelty are difficult to infer from the historical record, though, so creativity is often discussed but rarely measured (1, 2). In cultural evolution and cognitive science, creativity is usually understood as a search process that results in something “novel, surprising, and valuable” (3), which are not equally measurable. Novelty is tractable: a variant is novel to the extent that it occupies a less probable region of representational space (4). Value is domain-specific and ultimately depends on the judgments of other people. In this study, we measure novelty directly and treat the choices of other users—which images they like, and which they choose to build on—as partial evidence of value, since an image that is remixed has been selected by someone other than its creator (see Discussion).

Culture is a collective phenomenon, where individuals build on the ideas and behaviors of others (5), typically by recombining and modifying existing artifacts (6). A central paradigm for studying these cumulative dynamics is iterated learning, where content is passed along transmission chains and progressively shaped by learners’ biases (7). Iterated learning reliably produces simpler, more structured outputs that converge toward attractors shaped by cognitive and production biases, in systems ranging from artificial languages (7) to writing systems (8) to songs (9). Recent work has extended the paradigm in two directions: from linear chains to networks of interacting participants (10), and from purely human transmission to hybrid human–AI systems in which content is jointly created by people and generative models (11, 12). Digital creative platforms where users build on one another’s work with generative AI are naturally occurring instances of this setting and allow us to observe how cumulative culture unfolds when the transmission graph branches and the learners are humans collaborating with AI.

Artbreeder is a collaborative, AI visual art platform, originally based on BigGAN and StyleGAN (13, 14) and now using Stable Diffusion and FLUX (15, 16). These models take user-supplied images and text prompts as inputs and produce new images as outputs, so users can mix and modify existing content rather than generating from scratch (Fig. 1). Unlike other major platforms like Midjourney, Artbreeder emphasizes this kind of remixing (17). All recent Artbreeder tools let users iteratively combine multiple images and text prompts, adjusting the relative weight of each input. The four most heavily used of these tools—Collage, Composer, Mixer, and Splicer2—differ in the interface used to combine inputs, but all share the same basic structure of remixing one or more parent images and an optional text prompt into a single child image.

**Figure 1:**
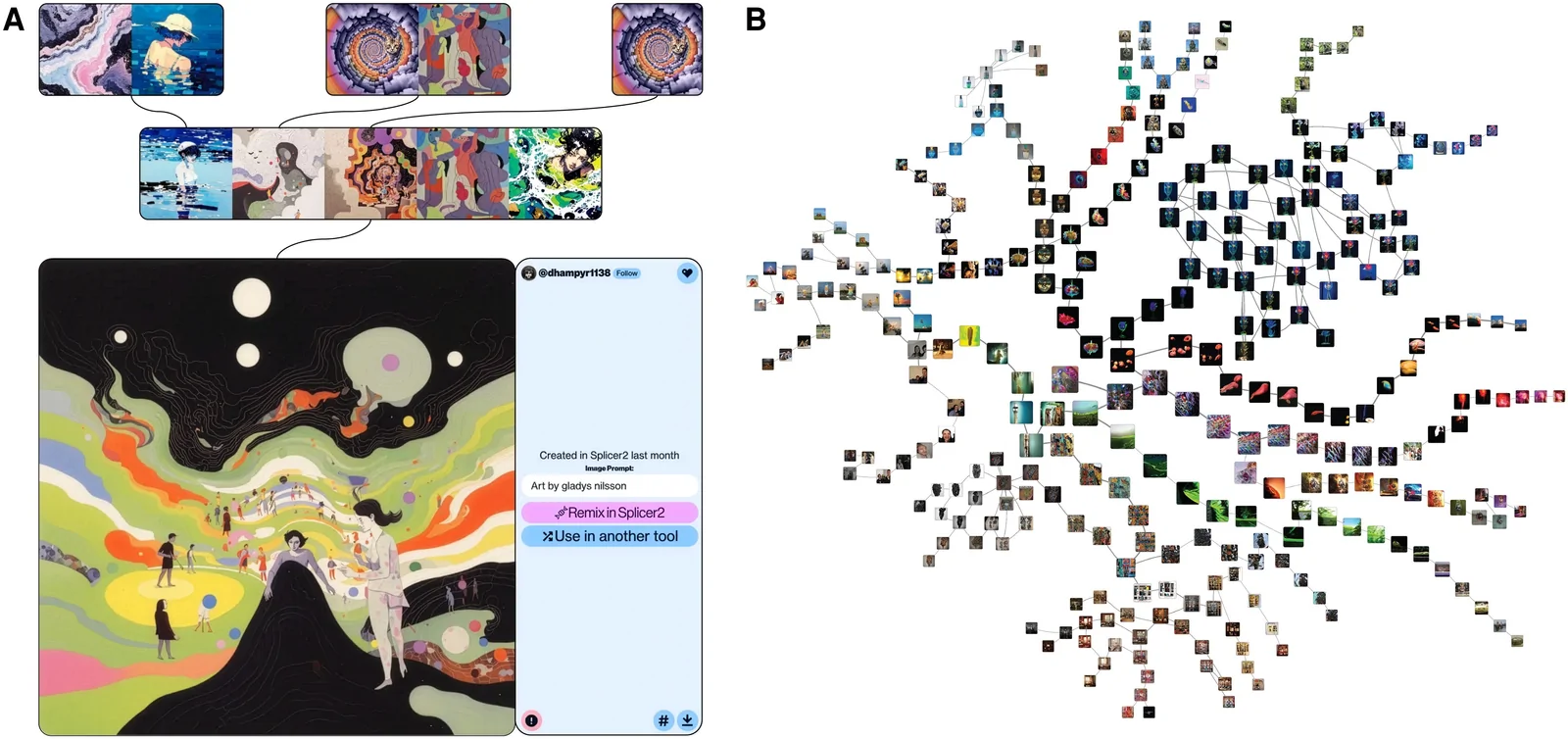
A) An example of an entry in an Artbreeder remix party, with its parents and grandparents above and the text prompt to the right. B) A print from Joel Simon’s *Latent Lineages* series, shown at Art Basel in 2025, depicting the branching lineages of remixes from a single image on Artbreeder.

Since mid-April 2024, Artbreeder has been hosting daily “remix parties” where the platform posts a seed image as the first entry, and users are asked to remix any image entered into the party, to “keep some aspect of the original while adding your own twist,” and to share the result. Lineages of remixes then branch out from the seed (Fig. 1). During each party, users see a timeline of others’ images and can inspect the generative process behind any of them (e.g. “user487 combined image351 and image713 with prompt316 to produce image906”). Users can like and comment on each image. Likes are far more common than comments (see Methods), so we use likes, along with whether an image is itself remixed, as our measures of “success.”

The aim of this study is to analyze a large corpus of remix parties from Artbreeder as a naturally occurring, networked, human–AI iterated learning system. Any cultural evolutionary process involves the inheritance of variation, its selection, and the structure of the population in which both occur (6), so we frame the analysis around three questions that map onto these components. First, how do the properties of parent images and text prompts shape the novelty and complexity of child images (inheritance and recombination)? Iterated learning shows that the fidelity with which content is transmitted constrains what can accumulate (7, 18), and models of cumulative culture treat the recombination of existing artifacts as a major source of innovation (6, 19). Every child here has known parents, and many draw on several at once, so we can ask whether combining dissimilar inputs injects novelty. Second, how do the properties of child images predict success in the form of likes and future remixes (selection)? A growing literature asks whether measurable features of cultural products predict their popularity, in paintings (20, 21), popular music (22, 23), and stories (24), as a window onto the biases that shape cultural evolution outside the laboratory. Here, we can ask whether novelty and complexity are favored in the same way by the users who like images and by those who remix them—a distinction that linear chains, where learner and selector are the same person, collapse. Third, how do population size, depth, and time within a party affect creative production (population structure)? Theory and experiments predict that larger groups sustain more complex and more diverse culture (25–28), but empirical tests are mixed (29, 30), and remix parties offer a clean comparison because they vary in size while sharing the same tools, seed structure, and duration. Classical iterated learning also predicts that lineages simplify and converge as transmission proceeds (7), which we can test using depth and time within a party. To answer these questions, we project all images and text prompts into a shared embedding space using OpenCLIP (31), calculate novelty and complexity using density estimation and image segmentation (32, 33), and use Bayesian multilevel structural equation models to estimate the relationships between variables (34).

## Methods

### Data

The data for this study come from all remix parties held between 2024 April 10 and 2025 May 5. We removed images from parties that lacked the seed image and images with missing data (e.g. an incomplete “key” in the database or no link to the seed), and only analyzed remixes produced with the four most heavily used tools that combine multiple inputs: Collage, Composer, Mixer, and Splicer2. After filtering, 130,882 images from 368 remix parties remained (∼77.5% of the original dataset).

To compute the density of images without bias, the density model has to be trained on different data than it is applied to, so we split the data into odd and even remix parties, trained on the odd parties, and only analyzed the even parties (see Novelty below). The modeled sample consists of the 65,108 nonseed images from the 184 even parties, produced by 780 creators using four tools (Composer: 31,913 images; Splicer2: 17,715; Collage: 10,082; Mixer: 5,398). Of these images, 67.9% were made with a text prompt and 62.8% had more than one parent image.

Users can also comment on images, but comments are too sparse to be used as an outcome: only 17,447 were posted in total, only 6.8% of modeled images received any (mean of 0.106), 26% were replies from the image’s own creator, and comment counts correlate with likes at *r* = 0.24. As such, we use likes and the number of grandchildren as outcomes and do not model comments.

Images on Artbreeder are in the public domain under a Creative Commons Zero license. The remix party data were exported from the Artbreeder database by J.N. as one JSON file per party, without any filtering (see Conflicts of interest and Data availability below). The backend generative models, the four tools, the ranking of the timeline, and the party interface were all stable over the sampling window. Time is coded within each party (see below) and every party and tool has its own random intercept, so calendar-level changes to the platform and differences between tools are absorbed by the intercepts rather than the time effects.

### Embeddings

Embeddings for images and text prompts were extracted with OpenCLIP (31), an open-source version of OpenAI’s CLIP that projects both images and text into a shared 1,024-dimensional space (Fig. 2). We used the CLIP-ViT-H-14-laion2B-s32B-b79K checkpoint (https://huggingface.co/laion/CLIP-ViT-H-14-laion2B-s32B-b79K).

**Figure 2:**
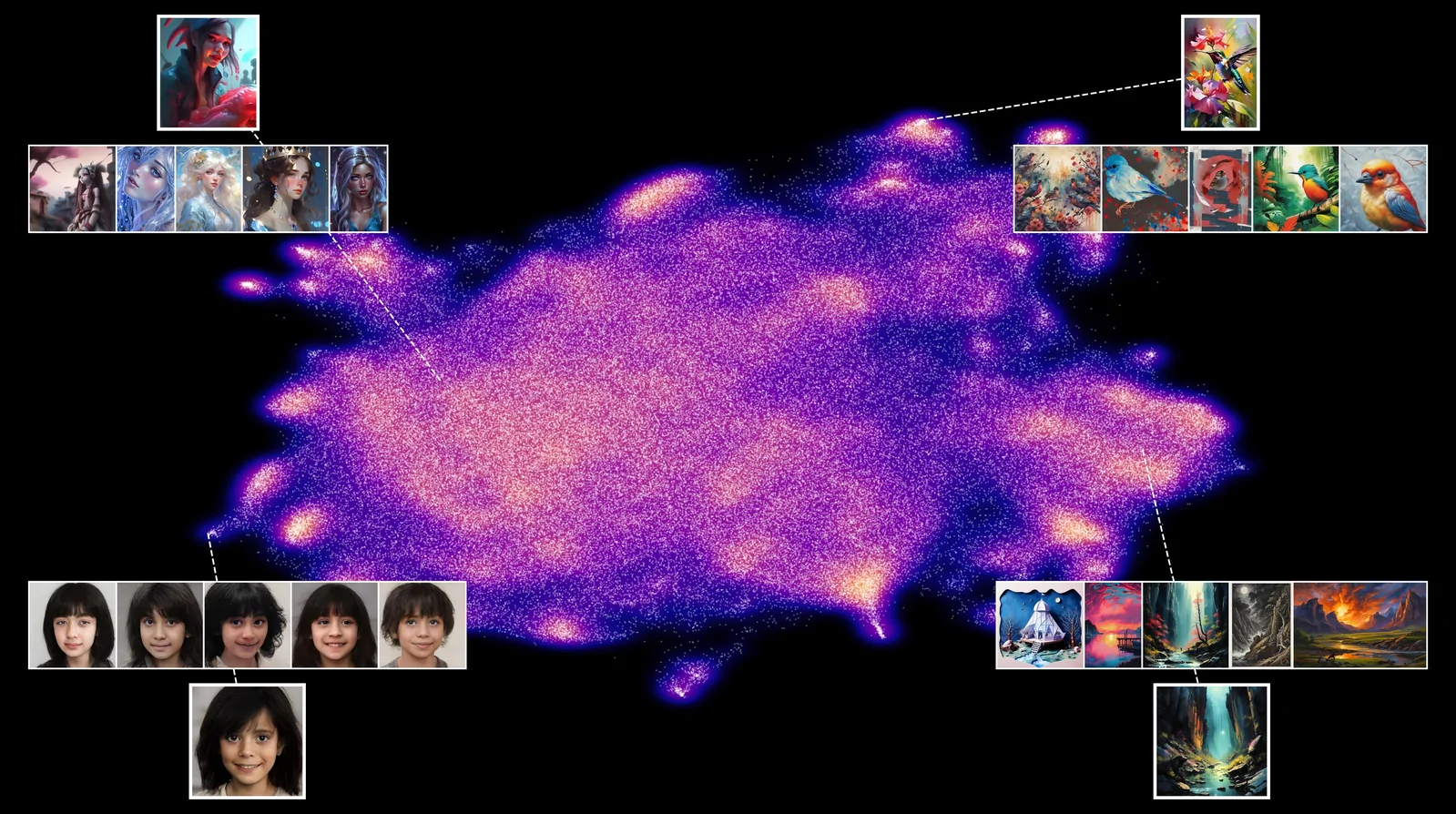
A UMAP projection of the OpenCLIP embedding space, with a kernel density estimate underneath. The projection is for visualization only, as the analysis is conducted in the raw embedding space with neural density estimation. The four large images are randomly chosen focal images, and the five smaller images next to each one are its nearest neighbors in embedding space.

### Distance

Distances between images and text prompts were computed using the cosine distance (1−cosinesimilarity) applied to the embeddings.

### Novelty

To compute the novelty of images and text prompts, we conducted density estimation using the *denmarf* package in Python (32). A single masked autoregressive flow was trained jointly on the image and text prompt embeddings from the odd remix parties. OpenCLIP places images and text in the same space, so a joint density describes what content in either modality is typical of the corpus. Embeddings were standardized before training, and the flow had 16 blocks with 1,024 hidden units, trained on 109,783 embeddings (80,102 images and 29,681 prompts) for 100 epochs with a batch size of 4,096, a learning rate of $10^{-3}$, and weight decay of $10^{-3}$, with an internal 80/10/10 train/validation/test split. The final training and validation negative log-likelihoods were −384.3 and −240.7 nats per sample. Validation loss was still decreasing slowly (about 0.7 nats per epoch, against a between-image spread of 230 nats) when training stopped, and the retained model is from the best-validation epoch (Fig. S1). Held-out log densities are unimodal for both images and prompts, which were z-scaled separately (Fig. S2). The fitted flow was then used to compute the log density of each image and text prompt from the even remix parties. Novelty was operationalized as the standardized negative log density under the flow, so an image (or prompt) in a low-density region of the embedding space is unusual relative to the corpus. Because the space is 1,024-dimensional, this quantity is a density rather than a probability mass, and only relative values are meaningful. The measure captures statistical atypicality rather than esthetic or semantic value. Images at the 10th, 50th, and 90th percentiles of novelty are shown in the Supplementary material (Fig. S3). The flow ranking is robust to a nonparametric alternative on the same embeddings: the mean cosine distance to the ten nearest training embeddings correlates at r=0.85 with flow novelty (Fig. S4). A 20-component Gaussian mixture on the first 50 principal components agrees only weakly (r=0.32), but this is a coarse comparison rather than a competing measure, as it retains 49% of the variance and cannot follow the curved, multimodal geometry of the space.

### Complexity

To compute the complexity of images, we counted the number of segments (i.e. groups of similar pixels) returned by FastSAM (33), a fast approximation of meta’s segment anything model (SAM) (35). We used the FastSAM-s checkpoint with an input size of 640 pixels, a confidence threshold of 0.05, and a maximum of 1,000 detections per image. Only 120 of the 65,108 modeled images (0.18%) reached this cap and one returned no segments, so censoring is negligible. Segment counts from SAM predict human ratings of visual complexity well across several image datasets (36). Complexity is a contested construct, though, with entropy-based (20) and compression-based (21) operationalizations, so we compared segment counts with the compressed (JPEG) file size of each image at a fixed resolution and with object counts from DETR (37). Log segment counts correlate at r=0.49 with log compressed size but only r=0.26 with log object counts (Fig. S4). DETR detects categories of everyday objects and returns zero to two detections for most of these images (median of 1, 32% zeros), so it is poorly suited to object-free art and cannot resolve differences between densely detailed but objectless images. Segment counts behave as a measure of visual detail, and images at the 10th, 50th, and 90th percentiles of segment count look as expected (Fig. S5). Character count was used as a coarse proxy of text prompt complexity.

### Depth, time, and population size

Three party-level variables enter the model. Depth is the number of remix steps between an image and the seed (range 1 to 19, median 3). Time is the number of seconds between the posting of the seed and the submission of the image, a within-party clock rather than a calendar date (median about 9 h). Population size is the number of unique creators who entered the party after filtering. Depth and time are moderately correlated (r=0.34). Images submitted later in a party have less time to accumulate likes and remixes before it ends, so the effects of time on the two outcomes partly reflect exposure rather than appeal.

### Structural equation modeling

Our model includes 15 variables related to the parent and child images, with dependencies between them (Table 1), so we built a multilevel structural equation model in Stan using the *brms* package in R (34), with the structure shown in Fig. 3. The graph follows the temporal ordering of remixing: the properties of the parent images and text prompt precede those of the child image, which precede the likes and remixes that the child receives, whereas party-level variables (population size, time, and depth) can affect all of them. The choice and ordering of variables follows from this and from the literature reviewed in the Introduction, rather than from a data-driven search over graph structures.

**Figure 3:**
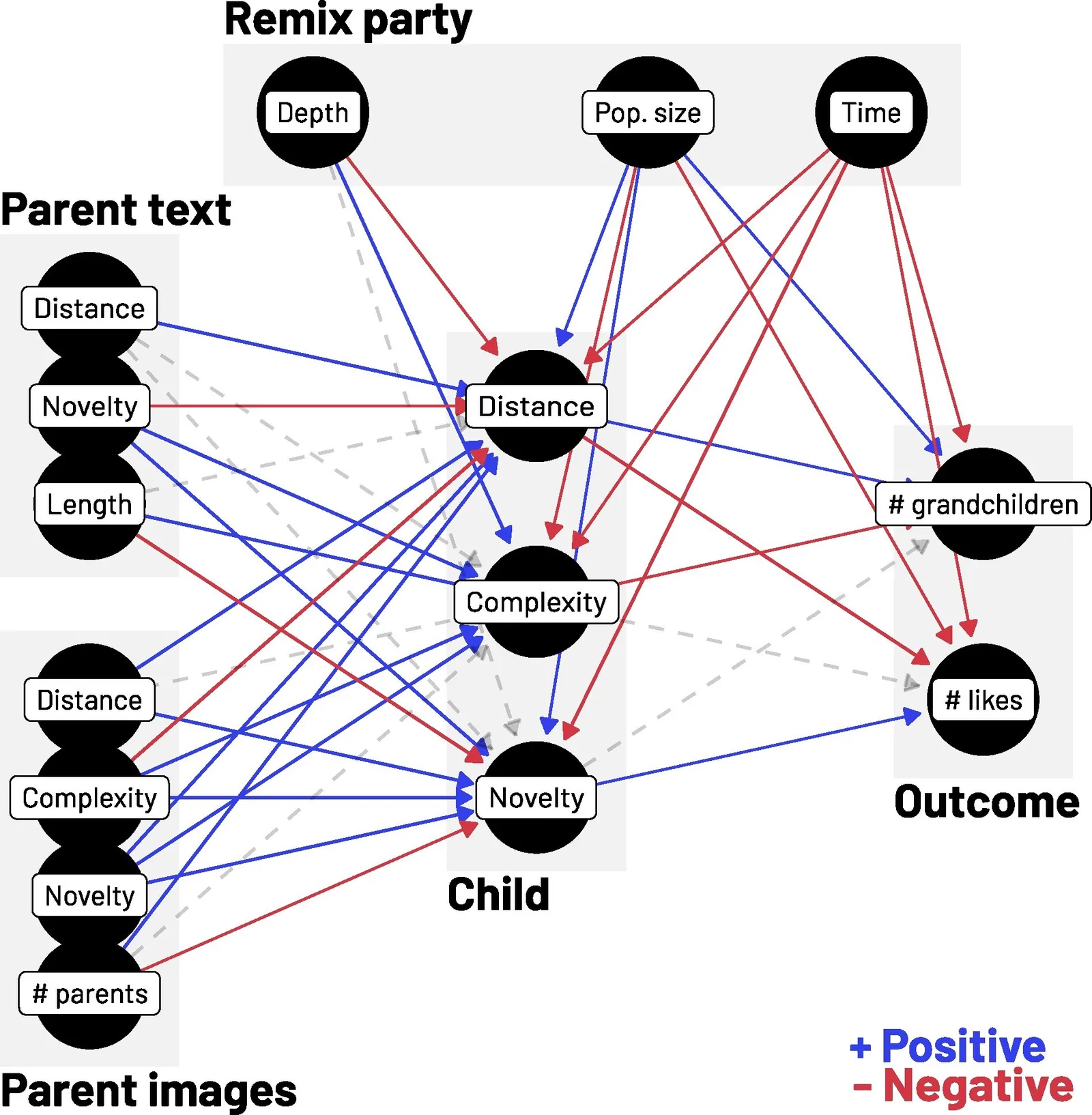
The structure and results of the Bayesian multilevel structural equation model, as a directed acyclic graph. Each node is a variable and each arrow is an assumed causal path. The color of each edge denotes the direction of the estimated conditional association along that path: blue is positive, red is negative, and dotted gray means that the 95% credible interval overlaps zero. The three child image equations are Gaussian, the number of likes is Poisson, and the number of grandchildren is negative binomial, so the five edges into the grandchildren node come from the negative binomial fit. Categories of variables are grouped in gray boxes (e.g. all measures of text prompts are in the top left).

**Table 1:** The variables included in the structural equation model.

Category	Variable	Missing data	Transform	Model family
Parent image	Distance (b/t parent images)	∼37.2%	z-scale	
Parent image	Complexity (no. of segments)		log and z-scale	
Parent image	Novelty (neg. log density)		z-scale	
Parent image	No. of parents		z-scale	
Parent image	Multiple parents (indicator)			
Parent text prompt	Distance (from parent images)	∼32.1%	z-scale	
Parent text prompt	Novelty (neg. log density)	∼32.1%	z-scale	
Parent text prompt	Length (no. of characters)	∼32.1%	z-scale	
Parent text prompt	Prompt used (indicator)			
Child image	Distance from parents		z-scale	Gaussian
Child image	Complexity (no. of segments)		log and z-scale	Gaussian
Child image	Novelty (neg. log density)		z-scale	Gaussian
Outcome	No. of grandchildren			Neg. binomial
Outcome	No. of likes			Poisson
Remix party	Depth (steps from seed)		z-scale	
Remix party	Population size (no. of creators)		z-scale	
Remix party	Time (since seed, within party)		z-scale	

Missing data correspond to cases with a single parent image (so no between-parent distance) or no text prompt. These cases are flagged by the two binary indicators, which enter the model through interactions (see below). Random intercepts for tool, remix party, and creator are included in every equation.

The model has five response equations. Each of the three child image measures (distance from parents, complexity, and novelty) is regressed on the number of parents, the complexity and novelty of the parent images, the distance between parent images, the length, novelty, and distance from the parent images of the text prompt, and population size, time, and depth. Each of the two outcome variables (number of likes and number of grandchildren) is regressed on the three child image measures, population size, and time. Because some tools do not require multiple parents or a prompt, the between-parent distance enters only through its interaction with a multiple-parents indicator, and the three prompt variables only through their interactions with a prompt-used indicator. Every equation includes random intercepts for tool (four levels), remix party (184), and creator (780), which absorb differences between tools, between parties (including calendar-level changes to the platform), and between users. Residual correlations between the five responses were fixed at zero, so the model is equivalent to five univariate multilevel regressions that share no parameters, which the sensitivity analyses take advantage of.

Besides the number of likes and grandchildren, all variables were z-scaled prior to modeling, and those with lognormal distributions were also log-transformed (Table 1). Seed images were excluded as children but included in the parent image measures. Missing data (Table 1) were handled with mean imputation after scaling, as multiple imputation was prohibitively expensive given the complexity of the model. Mean imputation ignores the fact that missingness here is structural (no second parent, no prompt), so the indicator interactions restrict these effects to cases where the variable is observed, and complete-case refits on the multiparent and prompted subsets can be seen in the Supplementary material (Tables S8 and S9). Imputation does not systematically attenuate the affected coefficients: the text prompt coefficients are nearly unchanged, whereas the between-parent distance coefficient on child novelty reverses sign (see Results).

Gaussian priors with mean 0 and SD 1 were used for all regression coefficients, and half-Gaussian priors with mean 0 and SD 0.5 for all group-level (random intercept) SD; intercepts and residual SD used the default weakly informative Student t priors in *brms*. The three child image measures were modeled as Gaussian, and the two outcome variables were modeled on their raw counts under different families: likes as Poisson and grandchildren as negative binomial (Table 1; see below). The model was fit with the *cmdstanr* backend using 15 chains of 2,000 iterations each (1,000 warmup) with adapt_delta  = 0.9; the grandchildren equation was fit on its own, with the same predictors, priors, and random intercepts, using four chains of 2,000 iterations.

We interpret the estimates as conditional associations under the assumed graph and random effects structure rather than as identified causal effects. Every parent of a response is a predictor in that response’s equation, so each direct path has a valid adjustment set made up of the other predictors in its equation. We verified with the *dagitty* package (38) that every direct path and total effect has such a set, although this check assumes that the graph is correct. Variables absent from the graph—a user’s skill and intentions, the semantic content of an image, how visible it was on the timeline—could bias any path, and the paths into likes and grandchildren depend on a selection process (which images users see and choose) that we do not observe.

### Model diagnostics and sensitivity analyses

Convergence diagnostics and posterior predictive checks for the main model are in the Supplementary material (Tables S2 and S3, Fig. S11). Both count outcomes are overdispersed relative to a Poisson (variance-to-mean ratios of 1.63 for likes and 4.97 for grandchildren, which are 64.7% zeros), so we fit each outcome under a Poisson, a negative binomial, and a hurdle negative binomial. When compared using approximate leave-one-out cross-validation and posterior predictive checks (Tables S4 and S5), the grandchildren equation clearly rejects the Poisson: its expected log predictive density is about 53 SE below the best-fitting family, it reproduces 1 of 9 predictive statistics, and it predicts 42.7% zeros against an observed 64.7%. The hurdle fits slightly better than the negative binomial (7 of 9 statistics against 6, but by only 0.019 nats per observation), but we take the negative binomial as the primary family because it reaches the observed share of zeros through overdispersion alone, without a separate process generating them, and because it keeps one coefficient per path in Fig. 3 rather than two. The hurdle is reported in the Supplementary material. Its shape parameter is 0.357 [0.349, 0.365], a conditional variance of about $\mu +2.8\mu^{2}$, and it converged well (maximum $\hat{R}=1.008$, 3 divergent transitions out of 4,000 draws, minimum bulk effective sample size of 936). Likes remain Poisson, as a Poisson already reproduces their zero share and their tail (see Results), and both negative-binomial-family fits of likes collapsed back towards it, leaving no residual overdispersion once the tool, party, and creator intercepts are included (Table S4). Two further specifications were refit as single equations with four chains of 2,000 iterations: the two indicators as main effects in the three child equations (Tables S6 and S7), and complete-case refits of the child equations on multiparent (n=40,879) and prompted (n=44,220) images (Tables S8 and S9).

## Results

Descriptive trends in the raw data (binned means of child novelty, complexity, and parent–child distance against depth and time within a party) and example lineages can be seen in the Supplementary material (Figs. S6, S7, and S8). The results of the Bayesian structural equation model can be seen in Fig. 3 and Table 2. We focus on five results:

1. More novel and more complex parent images produce more novel and more complex child images. In the full-sample model, the distance between parent images (i.e. how dissimilar they are from one another) also predicts higher child novelty but not child complexity, although this effect is not robust (see below).
2. More novel child images receive more likes.
3. More complex child images receive fewer future remixes (i.e. grandchildren), whereas more novel child images are neither remixed more nor less.
4. After accounting for the other predictors, images become less novel, slightly less complex, and closer to their parents as time within a party increases. In the raw data, novelty rises over the first 6 to 9 h and then falls by about 0.3 SD, parent–child distance declines, and complexity is flat (Fig. S7), so the negative time coefficient for complexity is conditional rather than a raw decline. Depth is associated with smaller parent–child distance and slightly higher complexity, but not with lower novelty. Parent–child distance falls monotonically with depth, from 0.45 at depth 1 to 0.30 at depth 13 and beyond, and declines from the early to the late third of a party in 85% of parties (Fig. S6). Whole-party dispersion falls from depth 3 onward in 73% of parties (it cannot fall earlier, because every depth-1 image is a child of the same seed). Clustering the embedding shows a small rise in concentration over a party—the Herfindahl index of the cluster shares increases by 0.0051 [0.0002, 0.0096], an interval that barely excludes zero—alongside a clearer shift in composition, described below (Table S1, Figs. S9 and S10).
5. Remix parties with larger populations (i.e. more participants) produce images that are more novel but less complex.

**Table 2:** The estimates from the Bayesian structural equation model outlined in Figure 3.

Outcome	Predictor	Est.	2.5%	97.5%	
Complexity (child)	No. of parents	0.005	−0.003	0.012	
Complexity (child)	Complexity (parent image)	0.442	0.436	0.449	*
Complexity (child)	Novelty (parent image)	0.052	0.046	0.059	*
Complexity (child)	Pop. size	−0.045	−0.061	−0.029	*
Complexity (child)	Time	−0.010	−0.016	−0.003	*
Complexity (child)	Depth	0.009	0.002	0.016	*
Complexity (child)	Distance (b/t parent images)	−0.001	−0.009	0.008	
Complexity (child)	Length (parent text)	0.075	0.064	0.086	*
Complexity (child)	Novelty (parent text)	0.042	0.034	0.051	*
Complexity (child)	Distance (parent text to parent images)	0.005	−0.002	0.013	
Novelty (child)	No. of parents	−0.130	−0.137	−0.122	*
Novelty (child)	Complexity (parent image)	0.057	0.051	0.063	*
Novelty (child)	Novelty (parent image)	0.457	0.450	0.463	*
Novelty (child)	Pop. size	0.022	0.009	0.035	*
Novelty (child)	Time	−0.010	−0.016	−0.004	*
Novelty (child)	Depth	−0.003	−0.009	0.003	
Novelty (child)	Distance (b/t parent images)	0.030	0.022	0.038	*
Novelty (child)	Length (parent text)	−0.037	−0.048	−0.027	*
Novelty (child)	Novelty (parent text)	0.253	0.245	0.262	*
Novelty (child)	Distance (parent text to parent images)	0.004	−0.004	0.011	
Distance (child)	No. of parents	0.192	0.186	0.199	*
Distance (child)	Complexity (parent image)	−0.007	−0.013	−0.002	*
Distance (child)	Novelty (parent image)	0.261	0.255	0.267	*
Distance (child)	Pop. size	0.054	0.041	0.067	*
Distance (child)	Time	−0.013	−0.019	−0.007	*
Distance (child)	Depth	−0.038	−0.044	−0.032	*
Distance (child)	Distance (b/t parent images)	0.347	0.340	0.354	*
Distance (child)	Length (parent text)	−0.001	−0.010	0.009	
Distance (child)	Novelty (parent text)	−0.024	−0.032	−0.017	*
Distance (child)	Distance (parent text to parent images)	0.510	0.503	0.517	*
No. of likes	Complexity (child)	0.002	−0.004	0.009	
No. of likes	Novelty (child)	0.040	0.034	0.047	*
No. of likes	Distance (parent to child)	−0.011	−0.017	−0.004	*
No. of likes	Pop. size	−0.241	−0.284	−0.202	*
No. of likes	Time	−0.238	−0.245	−0.232	*
No. of grandchildren	Complexity (child)	−0.069	−0.087	−0.051	*
No. of grandchildren	Novelty (child)	−0.002	−0.021	0.017	
No. of grandchildren	Distance (parent to child)	0.028	0.009	0.046	*
No. of grandchildren	Pop. size	0.040	0.013	0.067	*
No. of grandchildren	Time	−0.430	−0.447	−0.412	*

The three child image equations are Gaussian, the number of likes is Poisson, and the number of grandchildren is negative binomial (see Methods). Estimates whose 95% credible intervals do not overlap zero are marked with an asterisk.

The model accounts for much of the variance in complexity ($R^{2}=0.455$), novelty (0.496), distance (0.580), and likes (0.428), but little of the variance in grandchildren (0.072).

The sampler converged (maximum $\hat{R}=1.023$, minimum bulk and tail effective sample sizes of 625 and 1,273, no divergent transitions in 15,000 postwarmup draws), although about half of the iterations reached the maximum tree depth, an efficiency issue (Table S2). Posterior predictive checks support the two count families. A Poisson reproduces likes well (observed share of zeros 0.189, predicted 0.193 [0.190, 0.196]) but not grandchildren, where it predicts 0.427 [0.422, 0.432] zeros against an observed 0.647 and underpredicts both the maximum and the SD (Fig. S11). The negative binomial recovers all three (Table S4).

Across the specification checks (Tables S4 through S9), the paths from parent novelty and complexity to child novelty and complexity, the negative effect of complexity on grandchildren, and the negative effect of population size on complexity keep their sign, with an interval excluding zero, in every specification in which they appear. Two results are less robust. First, novelty has no clear association with the number of grandchildren (−0.002 [−0.021, 0.017]). The rejected Poisson returns a small negative coefficient (−0.012 [−0.022, −0.002]) only because its intervals are far too narrow, and the count part of the hurdle agrees with the negative binomial (0.001 [−0.023, 0.025]). Complexity, on the other hand, reduces grandchildren under every family (−0.053 to −0.070), and the parent–child distance and population size effects keep intervals excluding zero, although they are 30% and 47% smaller than their Poisson counterparts. Under the hurdle decomposition, novelty acts on whether an image is remixed at all rather than on how often: it raises the probability of receiving no remixes (0.027 [0.006, 0.047]), as does complexity (0.055 [0.035, 0.074]), whereas neither predicts the count among images that are remixed (Table S5). This refines where these effects sit rather than showing that novel images accumulate fewer remixes overall. Second, the positive coefficient of between-parent distance on child novelty (0.030 [0.022, 0.038]) is negative in the complete-case refit on multiparent images (−0.047 [−0.056, −0.038]), where the parent novelty slope is steeper and the two predictors are correlated (r=−0.56), so we do not consider it reliable. In that same subset, the positive population size effect on novelty also loses its interval (0.012 [−0.003, 0.027]). The indicator model changes no signs, moves two small coefficients across zero, shrinks the number-of-parents coefficient on novelty from −0.130 to −0.072, and leaves the outcome equations and $R^{2}$ unchanged (Table S6).

## Discussion

Based on the structural equation model, more novel and complex parent images produce more novel and complex child images—a form of transmission fidelity that mirrors classical findings from linear iterated learning chains (7, 18). The diversity of parent images (i.e. how spread out they are in embedding space) also predicts child novelty in the full-sample model, which would suggest that drawing on multiple, dissimilar ancestors injects variation into lineages, consistent with accounts of innovation that emphasize the recombination of existing artifacts (6, 19). This kind of effect cannot be detected in linear chains, which transmit from a single predecessor, but it is not robust here. It reverses sign in a complete-case refit restricted to multiparent images, the only subset in which between-parent distance is actually observed (Table S8), so we draw no conclusions about recombination.

More novel child images receive more likes but are not remixed more, and more complex child images are remixed less but are not liked more. In other words, the images that get liked are not the images that get remixed. This asymmetry should be interpreted with caution. Likes are a coarse signal of approval rather than a measure of esthetic preference, remix counts reflect which images users chose to build on rather than how they would rate them, and the same users may both like and remix, so we are unable to fully separate “consumer” and “producer” roles. That being said, classical iterated learning obscures even this coarse version of the asymmetry, because the “learner” and the “selector” are the same person. Networked platforms like Artbreeder make the two visible and suggest that the simplification seen in iterated learning chains may be driven as much by producers choosing easier-to-modify inputs as by individual-level transmission biases. An analogy is sampling in hip-hop and electronic music, where the most common samples are drum breaks—simple rhythmic sequences that are easy to modify (39). Two further points should be highlighted. First, the model explains little of the variance in the number of grandchildren ($R^{2}=0.072$), which is strongly overdispersed and zero-heavy, and is the reason that equation is a negative binomial rather than a Poisson. Second, under that family novelty is unrelated to how many remixes an image receives, although it does raise the chance of receiving none at all (Tables S4 and S5). As such, we are most confident in concluding that novelty attracts likes but not remixes, whereas complexity deters remixes but not likes.

We also found that images become less novel and closer to their parents over the course of remix parties, and that parent–child distance shrinks with depth, in the raw data and the model. Convergence is a core prediction of iterated learning, documented for artificial languages (7), writing systems (8), and songs (9), and it emerges here despite the branching, hybrid structure of remix parties. Shrinking parent–child distance is the signature that iterated learning studies use to demonstrate convergence, as changes from one generation to the next become smaller when lineages stabilize (7), and here each remix step moves less far from its parents than the step before in 85% of parties. Whether the images within a party also become more similar to one another is less clear, as whole-party dispersion falls once remixing has branched away from the seed, from depth 3 onward in 73% of parties, but not before. Two caveats should be highlighted. First, once time is accounted for, depth is not associated with lower novelty and is associated with slightly higher complexity, so the decline in novelty is a function of elapsed time within a party more than the number of remix steps. The negative time effect on complexity is also small and conditional, since the raw complexity trend over a party is flat (Fig. S7). Second, we have not demonstrated attractors in the strict sense of regions that lineages settle into and stop leaving. Clustering the embedding into 15 regions shows only a small increase in concentration, with an interval that barely excludes zero, but a clear shift in composition from scenes to figures. Of the clusters whose change in share has an interval excluding zero, all five that gain are ones in which a human figure dominates the frame—close-up portraits of women (+3.28 percentage points [+1.42,+5.18]), armored fantasy figures (+2.56), women in period gowns (+2.08), couples dancing or embracing (+1.92), and ornate crowned portraits (+1.38)—whereas all four that lose either contain no human figure (landscapes and architecture −5.12, flowers and abstract pattern −2.98, insects and aquatic organisms −0.70) or place a small figure inside a large scene (−0.94; Table S1, Fig. S10). The clusters that grow mostly depict young women in fantasy, period, and decorative styles. These labels describe what the images depict, were assigned by inspecting them and are not measured attributes of any person. We interpret this shift as a drift toward the dominant portrait style of this platform and user base rather than as evidence for thematic attractors, and it is weak relative to the stabilization of parent–child distance.

What makes this setting distinctive is that the “transmitter” is not just a human. Biases in the training data of the underlying generative models push remixes toward certain kinds of content (12), platform norms shape what users choose to build on, and human cognitive biases operate on top of both. This raises an alternative explanation for convergence. Generative models homogenize outputs on their own: Doshi and Hauser (40) found that access to generative AI raised the novelty of individual stories while reducing the collective diversity of what was produced, and the same could happen here if the underlying models pull remixes toward a limited set of well-represented styles regardless of what users select. A drift toward a dominant portrait style is exactly what this account predicts. We are unable to separate convergence driven by human selection from convergence driven by the models. Despite this, the persistence of classical iterated learning signatures in this hybrid setting suggests that a broader view of the “learner”—one that encompasses both cognitive and algorithmic biases—may be necessary for understanding cultural evolution in AI-mediated environments (41).

Finally, population size (i.e. the number of participants in a remix party) has a positive effect on novelty and a negative effect on complexity, and larger parties also produce children that are further from their parents. The novelty result is broadly consistent with work showing that larger populations sustain more cultural diversity and innovation (25, 26, 28), although the empirical literature is variable and includes counterexamples (30). The result for complexity is more puzzling. Population size has been associated with cultural complexity historically (27) and experimentally (26), but more recent work shows that it can sometimes inhibit complexity (29). Both effects hold across the refits (see Results). Our model does not explain the negative effect. It is not mediated by simplification accumulating along longer lineages, because depth has a small positive association with complexity once time is accounted for. We speculate that larger parties, where more entries compete for attention and per-image likes are diluted (population size has a strong negative effect on likes), may favor quicker and simpler remixes.

Several limitations of this study should be highlighted. First, all estimates are conditional associations under an assumed graph, and unmeasured variables such as user skill, intent, and the visibility of images on the timeline could bias them. Second, we lack detailed information about who participates in remix parties and why, so the patterns we observe may partly reflect platform-specific norms rather than general features of collective creativity. For example, the tendency for less complex images to receive more remixes could be strategic, with users gravitating toward what they know will be well received, rather than a cognitive bias. Similarly, remix party instructions ask users to “keep some aspect of the original while adding your own twist,” which may dampen the novelty of the children they produce. Finally, our measure of novelty captures statistical atypicality in representational space rather than creative value in the sense of Boden (3), so low-density images may be genuinely innovative or simply incoherent outputs of the underlying generative models. The examples in Fig. S3 bear this out. At the 10th percentile of novelty, the images are in the platform’s dominant style: polished painterly fantasy portraits of women, close to interchangeable. At the 90th, they are not broken but depart from that style in medium and genre—mock covers, cartoon interiors, poster landscapes, photoreal portraits—so what the measure treats as novel is largely a departure from the corpus’s dominant idiom rather than incoherence. The far tail is a different matter and does contain a small number of degenerate outputs. That more novel children attract more likes, and that every remixed image has been selected by another user, is partial evidence of value, but a more complete test would require perceptual ratings from human participants.

In summary, users of Artbreeder navigate a landscape of creative trade-offs. The images that get liked are not the images that get remixed: novelty attracts likes but not remixes, and simpler material is what users build on. The tendency of images to converge over time mirrors the classical findings of iterated learning experiments, even though transmission here is networked, driven by collective choice, and mediated by generative AI. Data from online platforms have transformed our understanding of large-scale cultural evolution, but the cumulative creative processes that generate novelty—and how they change when the learners are hybrid human-AI systems—remain understudied. Digital art platforms can fill this gap by acting as naturally occurring iterated learning experiments that bridge individual- and population-level cultural dynamics. As generative AI becomes a pervasive collaborator in cultural production, understanding these hybrid creative ecosystems will be essential for predicting how culture evolves.

## Supplementary Material

pgag369_Supplementary_Data

## Data Availability

The code for the main analysis, from processing the export through the models reported here, is available on GitHub at https://github.com/masonyoungblood/artbreeder. The original data (374 JSON files, one per remix party) are deposited on Zenodo (https://doi.org/10.5281/zenodo.22377042). Images on Artbreeder are released under a Creative Commons Zero license and can be retrieved from the Artbreeder content delivery network by the keys those files contain, so the full pipeline can be run from the deposited data.

## Preprints

This manuscript was posted on a preprint: https://arxiv.org/abs/2605.17141.
