# Supplementary material for: Dynamics of collective creativity in AI art competitions
Source: PNAS Nexus. 2026 Oct 23;5(11):pgag369. doi: 10.1093/pnasnexus/pgag369 (PMC13729119; doi:10.1093/pnasnexus/pgag369)
Supplement: Supplementary file 1 [file pgag369_supplementary_data.pdf]

Supplementary information for

Dynamics of collective creativity in AI art competitions

Mason Youngblood[1,*], Jeff Nusz[2] and Joel Simon[2]

[1]Institute for Advanced Computational Science, Stony Brook University, NY, USA
[2]Morphogen, CA, USA
[*]Corresponding author: masonyoungblood@gmail.com

Flow training and validation

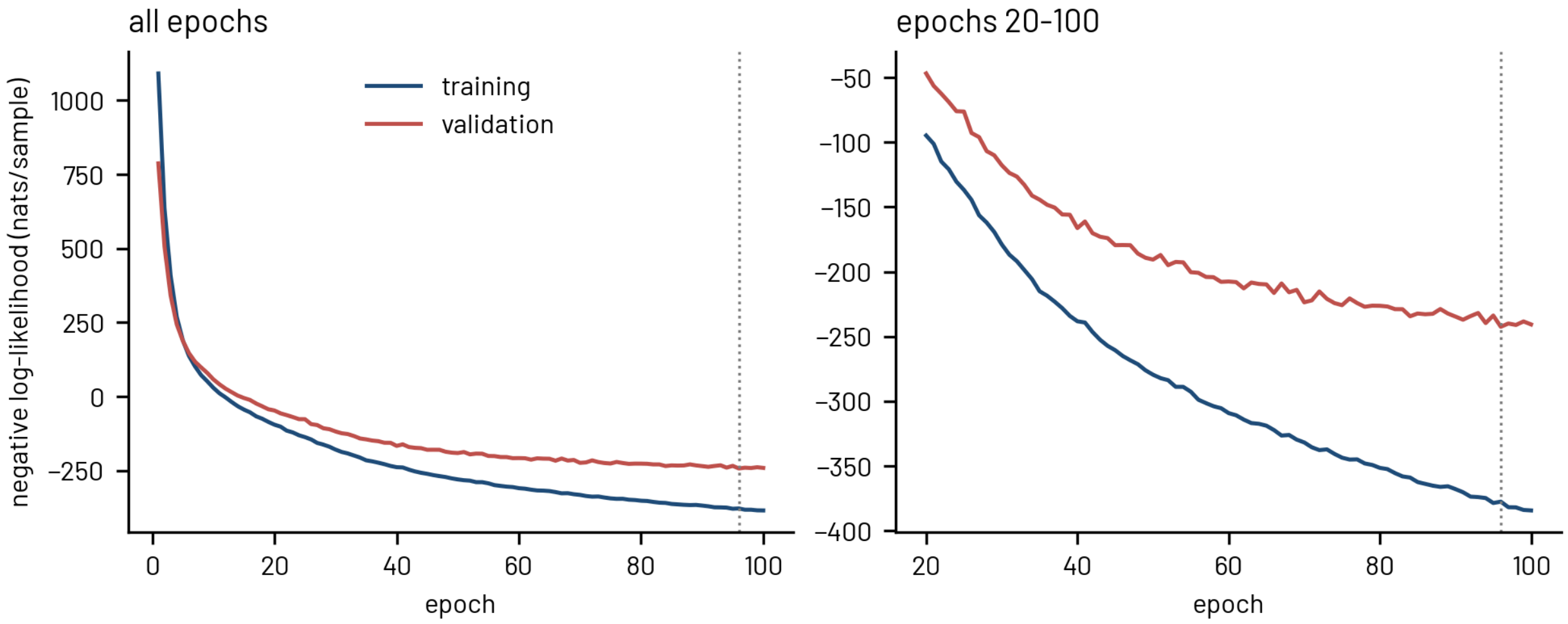

Figure S1: The training and validation negative log-likelihood (nats per sample) of the masked autoregressive flow across 100 epochs, for all epochs (left) and epochs 20 to 100 (right). Training loss fell from 1,091 to −384.3 and validation loss from 787 to −240.7. The dotted line marks epoch 96, where validation loss was lowest (−242.5) and from which the retained model comes. Validation loss was still decreasing at about 0.7 nats per epoch when training stopped, which is small relative to the 230-nat standard deviation of held-out image log densities. The 144-nat gap between training and validation loss is why novelty is only computed for parties that were not used in training.

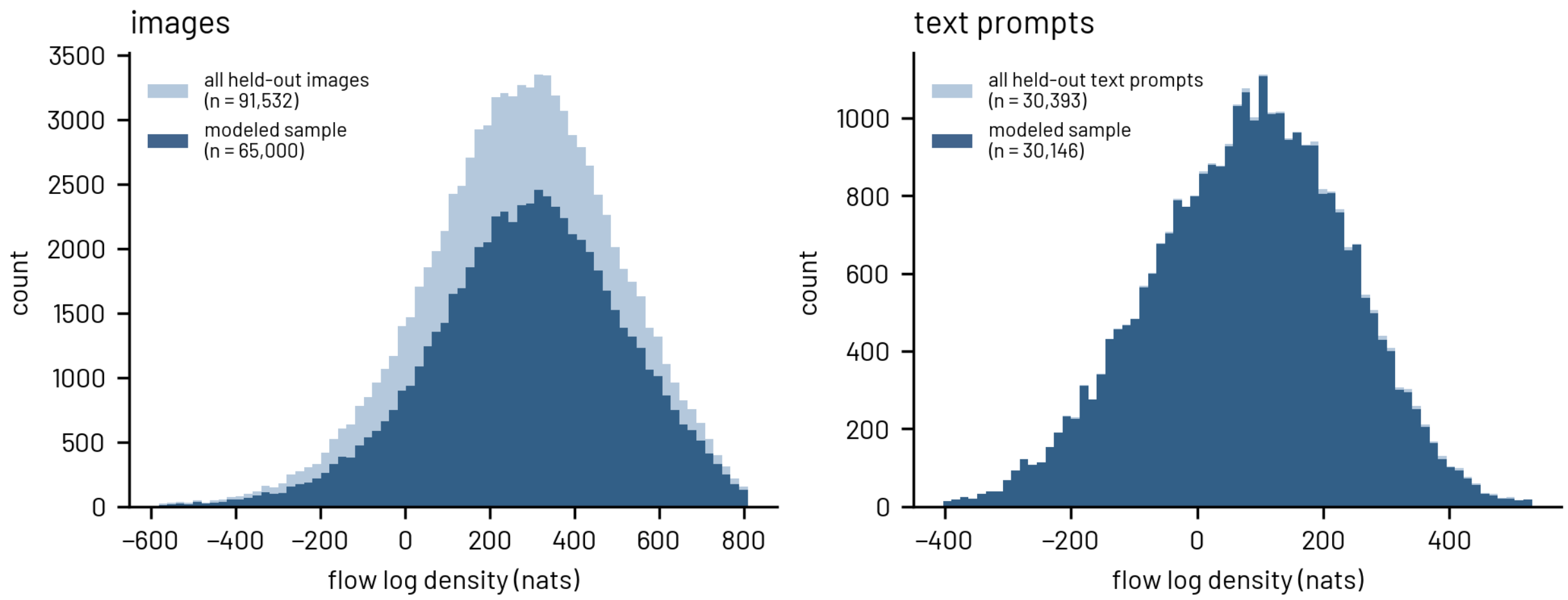

Figure S2: The log density under the fitted flow of all held-out images (left; n = 91,532, mean of 272, SD of 231) and text prompts (right; n = 30,393, mean of 82, SD of 159), with the modeled sample shaded (65,000 unique images with a mean of 285 and SD of 229; 30,146 unique prompts with a mean of 81 and SD of 159). Both distributions are unimodal and roughly symmetric, with no spike at either boundary. The offset of roughly 190 nats between images and prompts reflects the modality gap in CLIP and does not enter the models, because novelty is z-scaled separately for the two. One z unit of image novelty corresponds to 229 nats of log density.

Metric validation

Both metrics were compared with alternatives computed on the same 65,108 modeled images. The Pearson correlations are given in the Methods and in Figure S4, and the rank correlations match them (ρ = 0.85 for kNN novelty and 0.49 for log JPEG size). Of the 1% most novel images according to the flow, 41.7% are also in the top 1% according to the kNN measure. The 20-component Gaussian mixture agrees weakly whether its covariances are full (r = 0.32, retaining 48.8% of the variance) or diagonal (r = 0.26). JPEG (quality 90 at 512 by 512 pixels) and PNG sizes agree at r = 0.92.

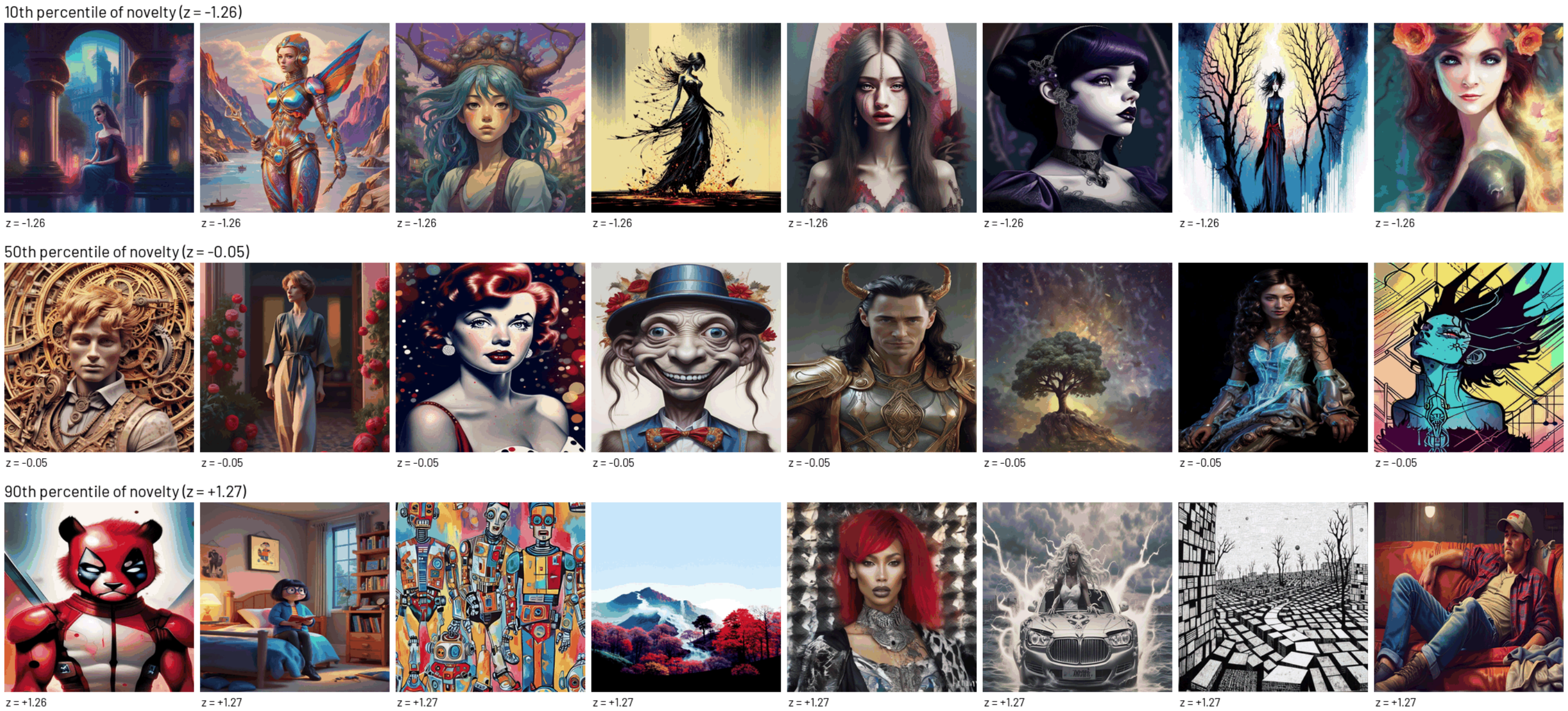

Figure S3: Eight images at each of the 10th, 50th, and 90th percentiles of novelty among the 65,108 modeled images, with the z-scaled score printed under each. At the 10th percentile ($z = -1.26$; log density of 574), the images are in the dominant style of the platform: polished painterly fantasy portraits of women that are close to interchangeable. At the 50th ($z = -0.05$), they are stylistically ordinary but visually heterogeneous. At the 90th ($z = +1.27$; log density of -5), they are not broken but depart from the dominant style in medium and genre: a mascot-style red panda, a cartoon bedroom interior, a folk-art crowd of robots, a poster landscape, photoreal portraits, and a monochrome surreal cityscape. Degenerate outputs sit far out in the tail rather than at the 90th percentile, and the single most novel image ($z = +6.75$) is an entirely black failed generation. Among the 30,146 unique prompts, the most novel are short, plain-English descriptions from outside the domain and the least novel are the boilerplate artist-tag strings of the platform.

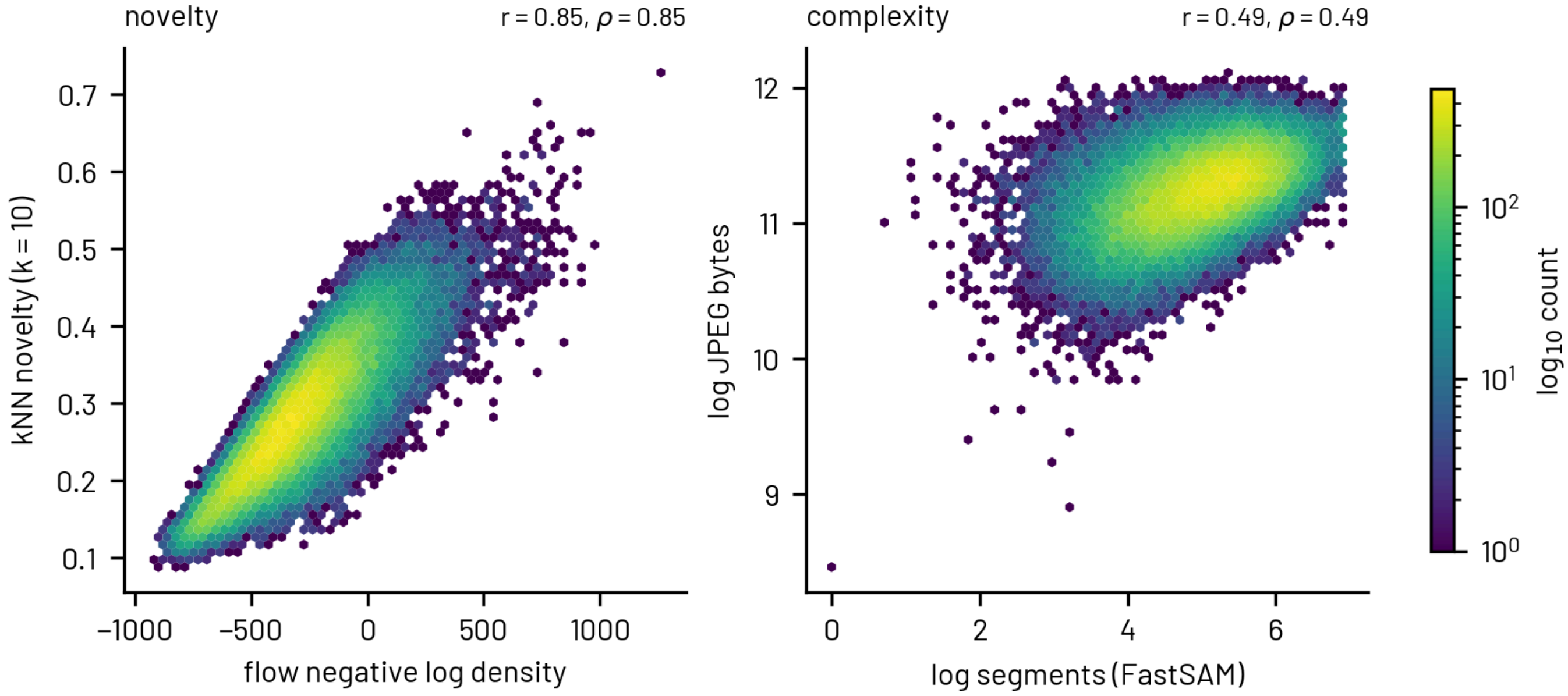

Figure S4: Each metric plotted against its main alternative for the 65,108 modeled images, as hexagonal bins on a logarithmic colour scale, with Pearson and Spearman correlations above each panel. The left panel shows flow negative log density against kNN novelty (k = 10; the mean cosine distance to the nearest training embeddings), which are nearly collinear (r = 0.85). The right panel shows log FastSAM segment count against log JPEG bytes at a fixed resolution (r = 0.49). The single point at zero segments in the right panel is the one image for which FastSAM returned no masks.

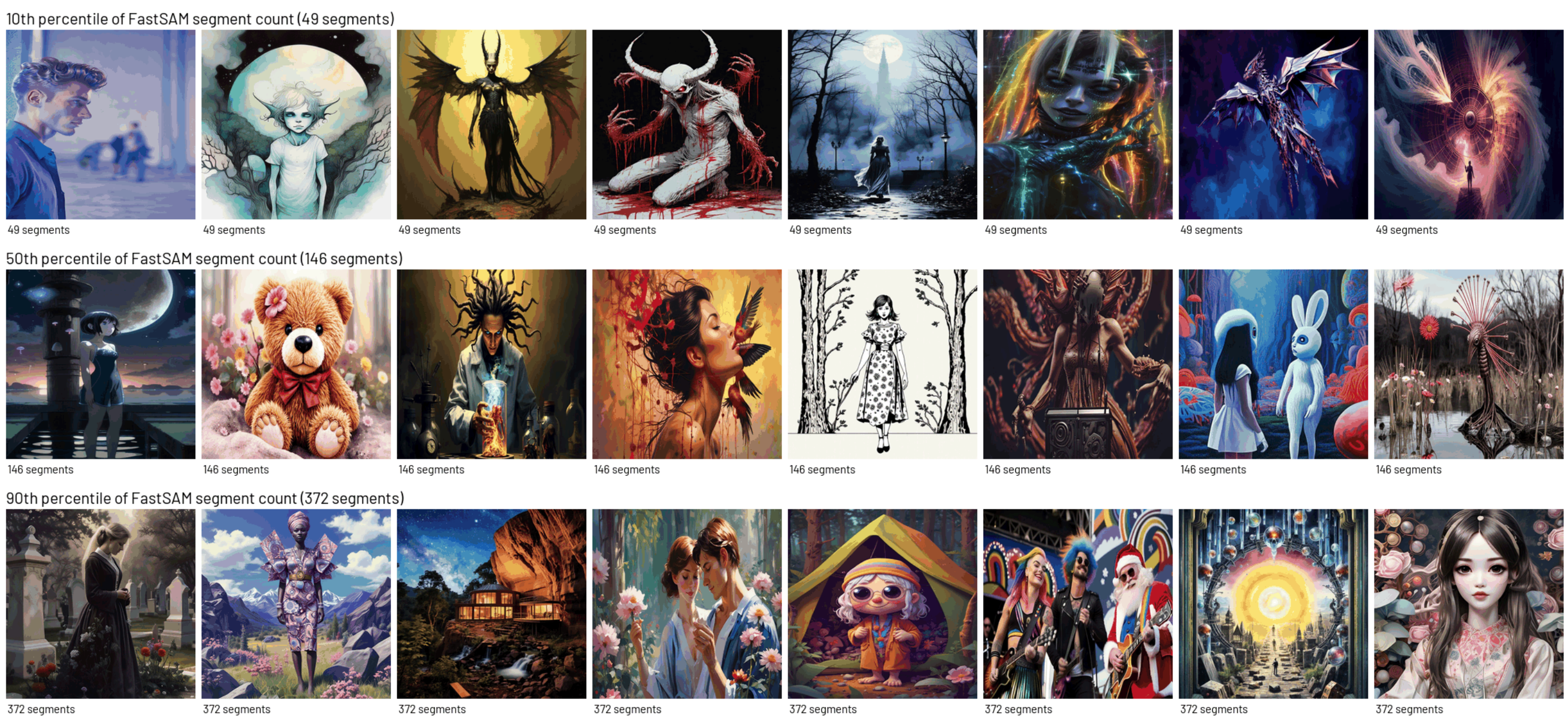

Figure S5: Eight images at each of the 10th, 50th, and 90th percentiles of FastSAM segment count, with the count printed under each. At the 10th percentile (49 segments), the images are sparse and low-contrast, mostly a single figure or silhouette against a dark or flat background. At the 50th (146), they are single subjects with a moderate amount of surrounding detail. At the 90th (372), they are dense and busy.

Descriptive trends

Every interval in this section and the next is a 95% percentile interval from a cluster bootstrap in which the 184 modeled parties are resampled with replacement (2,000 replicates), since images within a party share a seed, a pool of creators, and a theme.

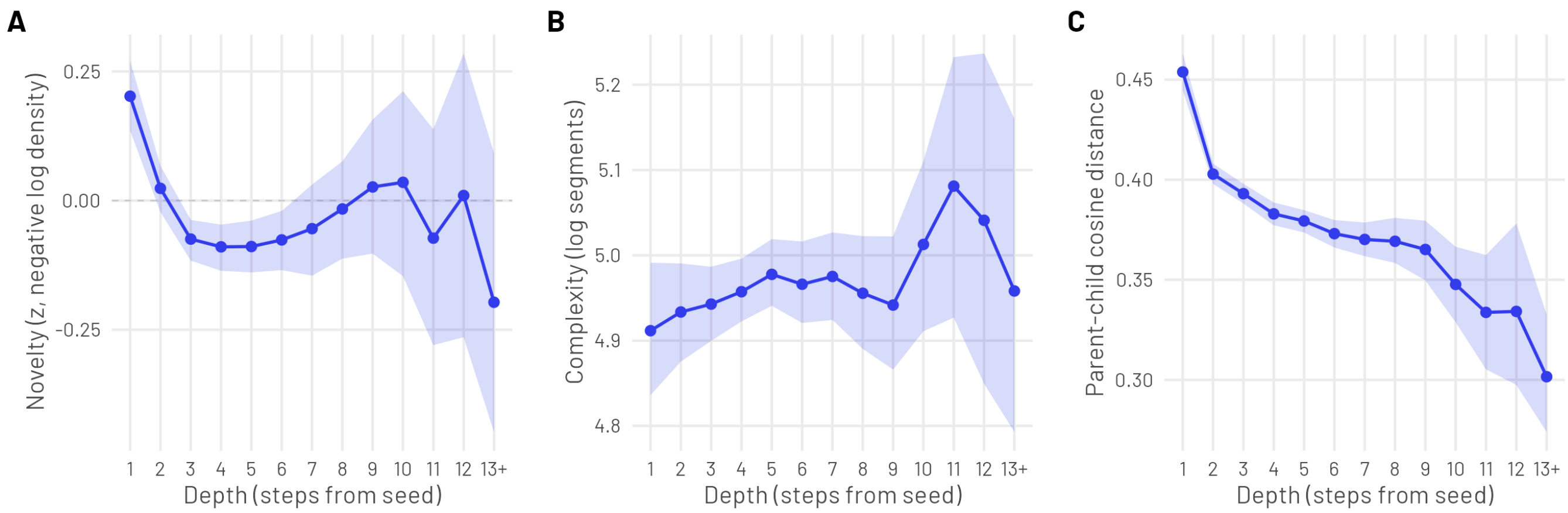

Figure S6: Raw trends in (A) child novelty (z-scaled negative log density), (B) child complexity (log segment count), and (C) parent-child cosine distance against depth, pooled across the modeled parties. Points are means at each depth from 1 to 12, with depths of 13 and above pooled (n = 12,379 at depth 1, 171 at 13+). Novelty is highest at depth 1 (0.20), falls through depths 3 to 5 (about −0.09), and is flat and noisy after that. Complexity rises slightly with depth (4.91 to about 5.01 log segments), with wide and overlapping intervals in the deepest bins. Parent-child distance falls monotonically, from 0.454 at depth 1 to 0.302 at depth 13+.

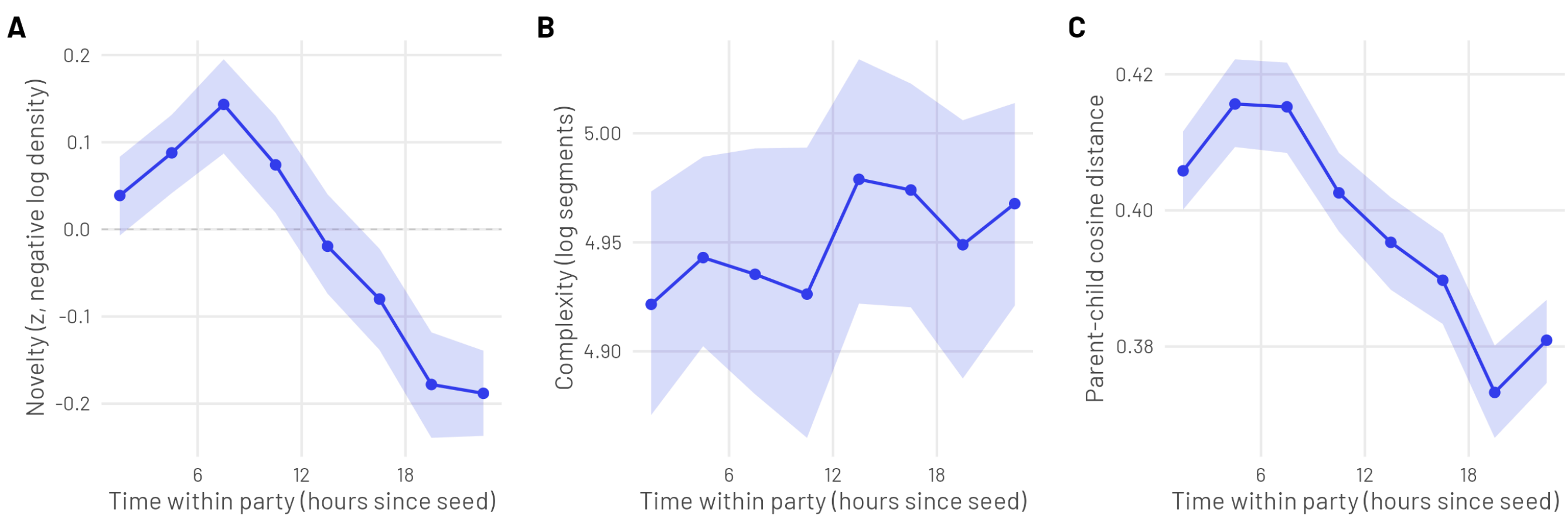

Figure S7: Raw trends in (A) child novelty, (B) child complexity, and (C) parent-child cosine distance against time within a party, in three-hour bins from the posting of the seed to the close of the party 24 hours later. The 20 images (0.03%) timestamped after the close are omitted from this figure only. Novelty rises for the first six to nine hours (+0.14 at 6 to 9 h) and then falls steadily to −0.19 in the final bin, a swing of about 0.3 standard deviations. Complexity is flat (4.92 to 4.98 log segments, with overlapping intervals throughout), so the small negative time coefficient for complexity in Table 2 is conditional rather than a visible raw decline. Parent-child distance declines from about 0.42 at 3 to 9 h to 0.37 to 0.38 in the last two bins.

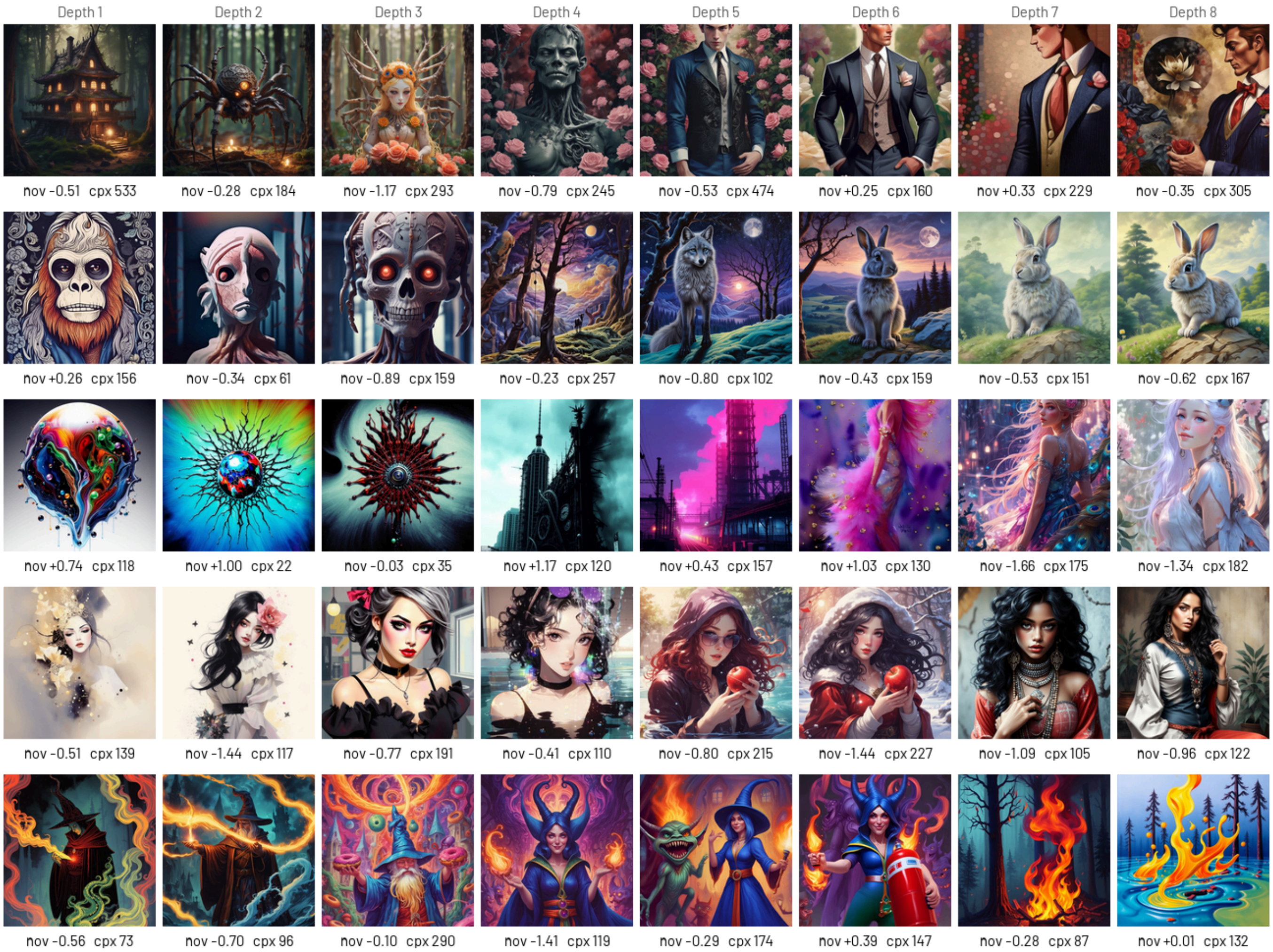

Figure S8: Five example remix chains, one from each of parties 230, 70, 349, 358, and 347, followed over depths 1 to 8 for a total of 40 modeled images. Seed images are not shown, and each step follows one parent within the same party. Novelty (z-scaled negative log density, "nov") and the raw FastSAM segment count ("cpx") are printed beneath each image, and the header row gives the depth of each column. Across all 40 images, novelty ranges from −1.66 to +1.17 and segment counts from 22 to 533. The five chains were chosen to be far apart in the embedding space (mean pairwise UMAP distance between chains of 2.62, against 1.98 for a uniform draw), so this is an illustration rather than a random sample. Averaged over the five chains, mean parent-child cosine distance falls from 0.418 over the first two steps to 0.317 over the last two, but only rows 2, 3, and 4 show that decline (most strongly row 2, from 0.548 to 0.103), whereas rows 1 and 5 run the other way (0.376 to 0.407 and 0.320 to 0.501). The stabilization reported in the Results is an average over 184 parties and is not visible in every lineage.

Convergence and attractors

Convergence was quantified using the L2-normalized OpenCLIP embeddings of the modeled sample. Dispersion is the mean pairwise cosine distance among all of the images in a party at a given stage, where stages are within-party depth terciles by rank (early: n = 28,470, mean depth of 1.6; mid: 19,594, 3.6; late: 17,044, 6.3), and every contrast is paired within parties.

Dispersion does not fall from the early to the late tercile (+0.011 [+0.002, +0.020]; 44% of parties decline). Early dispersion is necessarily low, because 12,188 of the 28,470 early images sit at depth 1 as children of the same seed, and it must rise as the tree branches. Against absolute depth, dispersion peaks at depths 2 to 3 and then falls by 0.046 [0.031, 0.062] by depth

8, in 73% of parties. Mean parent-child cosine distance falls from 0.421 [0.415, 0.428] in the early tercile to 0.367 [0.363, 0.372] in the late tercile (−0.054 [−0.061, −0.046]), in 85.2% of parties.

To examine attractors, we clustered the two-dimensional UMAP coordinates of the 65,108 modeled images with BIRCH (subcluster radius of 0.1 in UMAP units, default branching factor), so that the clusters describe the map shown in the figures. The number of clusters was set using the elbow of the within-cluster sum of squares, re-cut for every k from 5 to 60. The elbow is shallow—it lands at k = 15, but the criterion varies by only 4% across k = 13 to 18—so 15 is a choice of resolution. The compositional result does not depend on this choice (the same nine clusters change, with intervals excluding zero, at every k from 13 to 20), but the concentration statistics do, which is why the Results quote the Herfindahl index rather than the top-five share, whose early-to-late change ranges from +0.35 to +9.49 percentage points over that range.

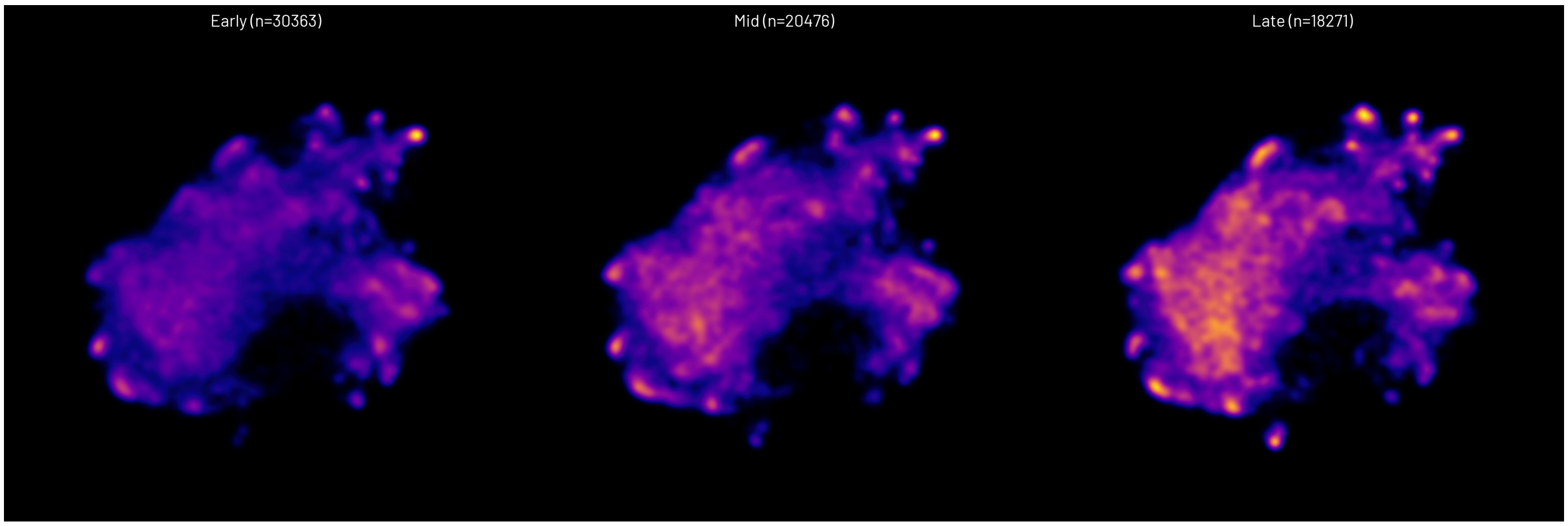

Figure S9: The density of images in the early, middle, and late depth terciles of their parties, as a UMAP projection of the OpenCLIP embedding space. Unlike the analyses above, this figure uses all 368 parties, including the seeds and the odd (training) parties (n = 30,363, 20,476, and 18,271). Colour is the logarithm of the binned count rather than a normalized density, so a stage with fewer images should appear dimmer, and yet the late panel has the fewest images and the brightest core. The projection is for visualization only, and the quantitative claims come from the text above and Table S1.

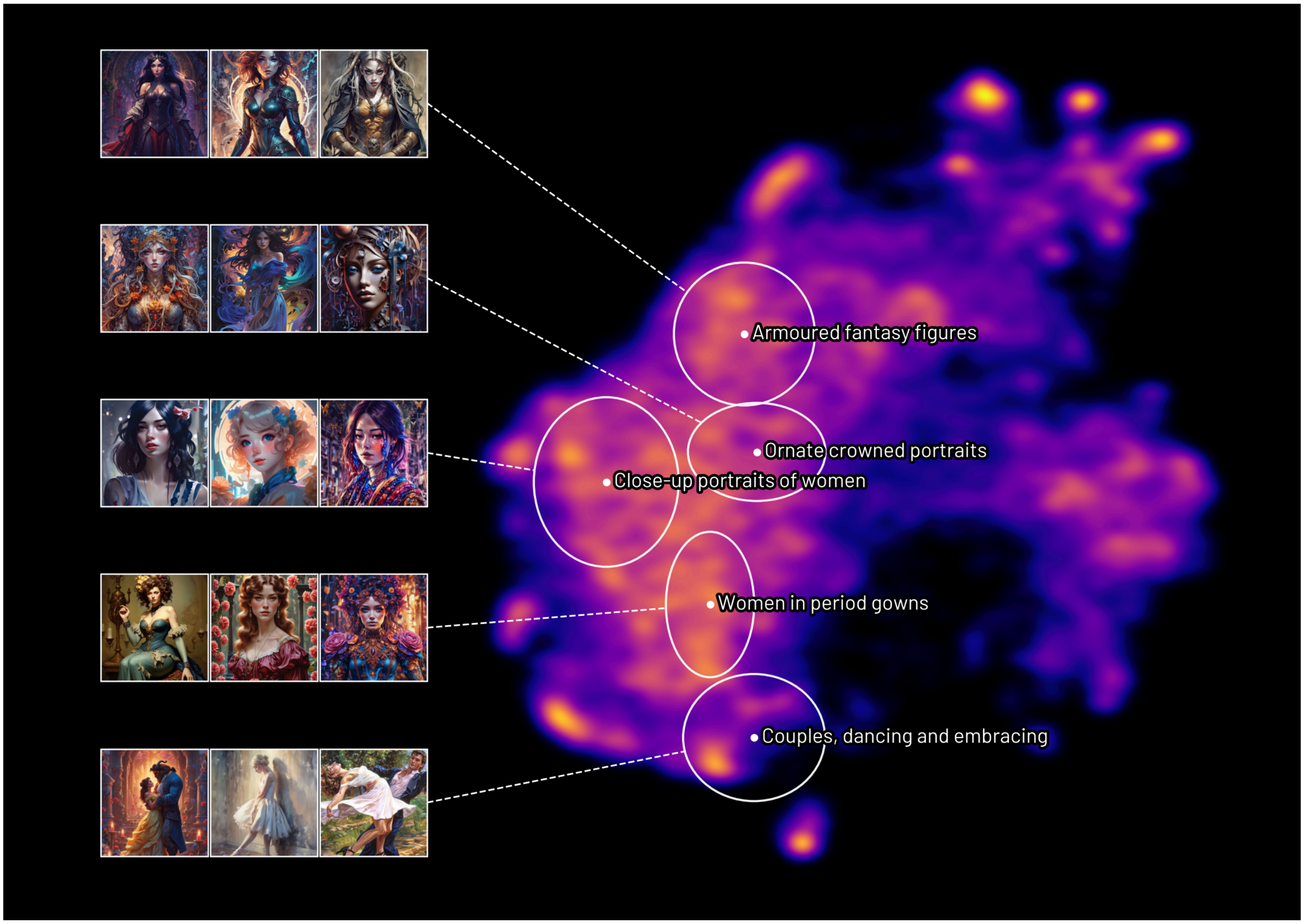

Figure S10: The UMAP projection of late-stage images only (the deepest within-party depth tercile; n = 17,044 modeled images), in the same format as Figure 2: a kernel density estimate with callout thumbnails of representative members joined to the map by dashed lines. The white rings mark 1.5 standard deviations of the members of the five BIRCH clusters (out of fifteen) whose share increased from the early to the late tercile with an interval excluding zero. Their early-to-late shares are: close-up portraits of women, 12.96% to 16.24% (+3.28 percentage points [+1.42, +5.18]); armoured fantasy figures, 9.01% to 11.56% (+2.56 [+1.08, +4.03]); women in period gowns, 7.10% to 9.18% (+2.08 [+0.87, +3.35]); couples dancing or embracing, 2.29% to 4.21% (+1.92 [+0.75, +3.26]); and ornate crowned portraits, 6.90% to 8.28% (+1.38 [+0.47, +2.35]). All five rings sit on the bright left lobe, the region that is brighter in the late panel of Figure S9 than in the early panel. Individual cluster members are not plotted, as 17,000 points would obscure the density. The cluster labels describe what the images depict and were assigned by inspecting them, and they are not measured attributes of any person.

Cluster	n	Early	Late	Change (pp), 95% CI	What the images depict
C1	9,462	12.96%	16.24%	+3.28 [+1.42, +5.18]	close-up portraits of single figures, almost all young women
C6	6,619	9.01%	11.56%	+2.56 [+1.08, +4.03]	armoured and costumed fantasy figures, mostly women
C11	5,304	7.10%	9.18%	+2.08 [+0.87, +3.35]	women in period gowns, interiors and gardens
C4	1,962	2.29%	4.21%	+1.92 [+0.75, +3.26]	two-figure scenes, couples dancing or embracing
C12	4,928	6.90%	8.28%	+1.38 [+0.47, +2.35]	ornate decorative portraits with crowns and headdresses
C5	5,415	8.05%	8.84%	+0.78 [−0.74, +2.32]	painterly period and contemporary portraits, women and men
C0	2,324	3.17%	3.86%	+0.69 [−0.06, +1.44]	full-length women in flowing gowns, water and moonlight settings
C14	3,538	5.64%	5.39%	−0.25 [−1.29, +0.74]	skulls, androids and macabre faces
C10	3,093	5.22%	4.48%	−0.74 [−2.12, +0.49]	anthropomorphic animals, foxes and cats
C7	3,378	5.52%	4.62%	−0.90 [−2.11, +0.19]	winged figures, angels, valkyries and demons

Cluster	n	Early	Late	Change (pp), 95% CI	What the images depict
C9	4,357	7.10%	6.04%	−1.06 [−2.62, +0.31]	fantasy creatures, beasts and dragons
C13	1,664	2.85%	2.15%	−0.70 [−1.36, −0.03]	insects, aquatic and alien organisms
C2	2,650	4.53%	3.59%	−0.94 [−1.81, −0.13]	small or silhouetted figures in large cosmic and atmospheric scenes
C8	3,313	6.51%	3.53%	−2.98 [−4.43, −1.64]	flowers, plants and abstract pattern and light effects
C3	7,101	13.15%	8.03%	−5.12 [−7.21, −3.01]	landscapes, ruins, buildings and portals, no dominant figure

Table S1: The share of images at each within-party stage that fall in each of the 15 BIRCH clusters, with the early-to-late change in percentage points and a 95% cluster-bootstrap interval over the 184 modeled parties. Clusters are grouped by whether that interval excludes zero: five gain share (top), six have intervals that cover zero (middle), and four lose share (bottom). Every cluster that gains share is one in which a human figure dominates the frame, and in four of the five that figure is predominantly a young woman in a fantasy, period, or decorative style. Every cluster that loses share either contains no human figure (C3, C8, C13) or places a small or silhouetted figure inside a large scene (C2). The same nine clusters change, with intervals excluding zero, at every k from 13 to 20. The descriptions in the last column are of depicted image content, arrived at by inspecting the ten images nearest to the centroid of each cluster together with twelve randomly drawn members. They are not measured attributes of any person, and each cluster is broad. A direct embedding probe of two candidate themes—the mean cosine similarity of party images to the average embedding of the 723 prompts containing "steampunk" and the 1,356 containing "alien"—shows no growth in either from early to late (steampunk: +0.004 [−0.001, +0.010]; alien: −0.004 [−0.009, +0.002]).

Model diagnostics

Quantity	Value
Post-warmup draws	15,000
Max Rhat	1.0233
Min bulk ESS	625
Min tail ESS	1,273
Divergent transitions	0

Table S2: Sampler diagnostics for the main model, fit with 15 chains of 2,000 iterations (1,000 warmup). There were no divergent transitions. About half of the iterations reached the maximum tree depth, which is an efficiency cost rather than a threat to the validity of the draws.

Outcome	Bayes R2	2.5%	97.5%
Complexity (child)	0.455	0.450	0.459
Novelty (child)	0.496	0.492	0.500
Distance (child)	0.580	0.577	0.583
# likes	0.428	0.422	0.435
# grandchildren	0.072	0.065	0.078

Table S3: The Bayes R^2 of the five response equations, with 95% credible intervals. The first four are from the main model, and the grandchildren row is from the negative binomial fit, which is the primary family for that outcome. The negative binomial explains less of the variance in grandchildren than the Poisson did (0.090 [0.087, 0.093]), because its residual variance includes an overdispersion term that the Poisson has no way to represent.

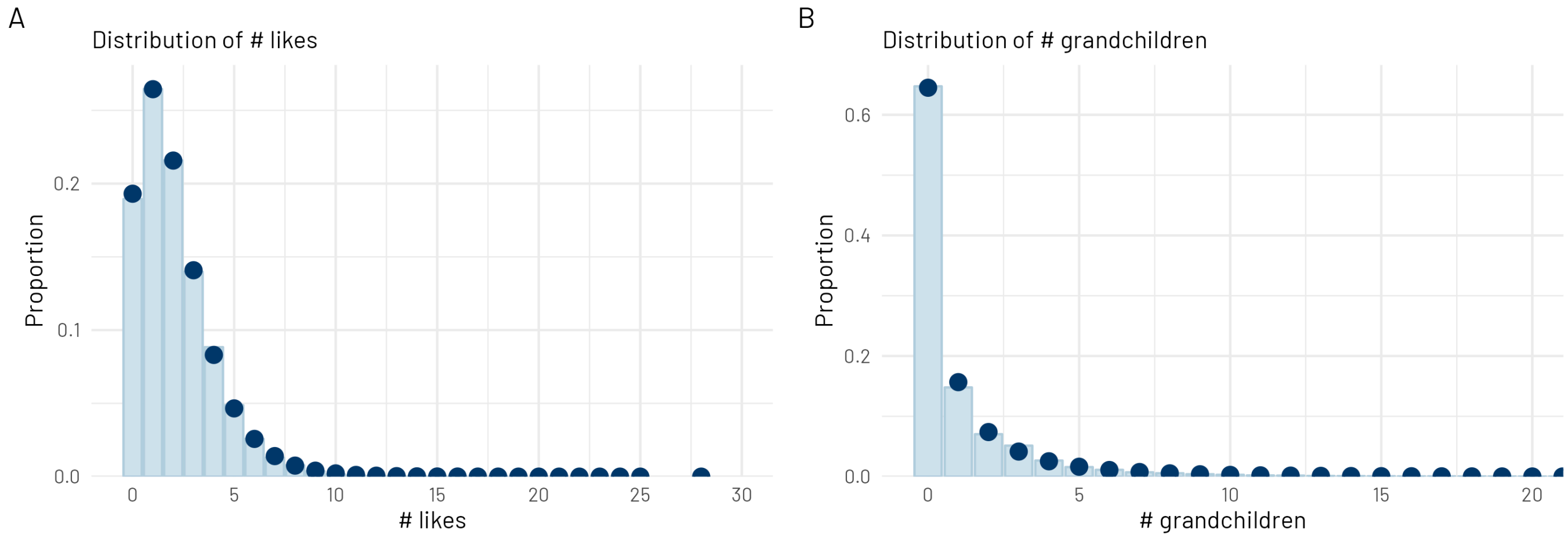

Figure S11: Posterior predictive checks for the two count outcomes, from posterior draws of the reported models: (A) likes under a Poisson likelihood and (B) grandchildren under a negative binomial. In both panels, the pale bars denote the observed proportion of images at each count and the dark points denote the replicated proportions from the posterior predictive distribution, with 95% intervals that are narrower than the plotting symbol at every count. Both families track the observed distribution closely, including the share of zeros (likes: 0.189 observed against 0.193 [0.190, 0.196] predicted; grandchildren: 0.647 against 0.645 [0.640, 0.650]). The full comparison of families can be seen in Table S4. The Poisson fit of grandchildren that is rejected there predicts only 0.427 [0.422, 0.432] zeros and underpredicts both the maximum (17.7 [13, 29] against an observed 51) and the standard deviation (1.20 against 2.23).

Sensitivity analyses

All sensitivity models are single-response *brms* fits of one equation, with the same transformations, mean imputation, priors, and random intercepts as the main model, run with four chains of 2,000 iterations (1,000 warmup) and `adapt_delta` = 0.9. Coefficients involving novelty are sign-flipped as in Table 2. The hurdle negative binomial has the same predictors and random intercepts in both parts, and Table S4 shows its count part while Table S5 shows both. The complete-case models keep the full-sample z-scores and use no imputation. Every fit tabulated below converged (maximum $\hat{R}$ = 1.017, at most 78 divergent transitions out of 4,000 draws).

Count families for the two outcomes

The grandchildren equation was fit as a Poisson (the `children` block of the main model), a negative binomial, and a hurdle negative binomial, on the same 65,108 observations. Approximate leave-one-out cross-validation (PSIS-LOO) ranks them in the order of hurdle negative binomial (expected log predictive density of −80,558), negative binomial (−81,796), and Poisson (−111,444). The negative binomial beats the Poisson by 29,648 (SE of 577), the hurdle beats the Poisson by 30,887 (SE of 583), and the hurdle beats the negative binomial by only 1,239 (SE of 50), or 0.019 nats per observation. The Poisson is also the only family with unreliable Pareto-*k* diagnostics. Of nine posterior predictive summary statistics (from 500 draws per family), the Poisson covers 1, the negative binomial 6, and the hurdle 7. The observed zero share of 0.647 is predicted as 0.427 [0.422, 0.432], 0.645 [0.640, 0.650], and 0.647 [0.642, 0.652], the observed standard deviation of 2.23 as 1.20, 2.27, and 2.18, and the observed maximum of 51 as 17, 60, and 51.

The Poisson is therefore ruled out. We chose the negative binomial as the primary family because it reaches the observed share of zeros through overdispersion alone and keeps one coefficient per edge in Figure 3, whereas the hurdle needs two and makes its count part conditional on being remixed at all. Both parts of the hurdle can be seen in Table S5, which is the source for statements about images that receive no remixes at all. Note that in *brms* 2.23.1, `posterior_predict` for hurdle negative binomial models draws the positive part with a zero-truncated Poisson correction, which biases the draws at small shape values, so the checks above use an exact inverse-CDF zero-truncated negative binomial sampler instead.

Likes remain Poisson. A Poisson reproduces their zero share (0.189 observed, 0.193 [0.190, 0.196] predicted) and their tail (Figure S11), and both negative-binomial-family fits of likes are degenerate, with the shape parameter unbounded above. All four chains of the plain negative binomial froze at single shape values between 2.8×10^7 and 1.5×10^9 ($\hat{R}$ undefined, bulk effective sample size of 4), and the hurdle reached a posterior median shape of 4.3×10^{12} with a maximum $\hat{R}$ of 2.88. In other words, the negative binomial collapses to the Poisson, leaving no residual overdispersion in likes once the party, creator, and tool random intercepts are included. No coefficients from either fit are quoted, and Table S4 covers the grandchildren equation only.

Predictor	Poisson (rejected)	Neg. binomial (primary)	Hurdle NB (count part)
Complexity (child)	−0.070 [−0.079, −0.061]*	−0.069 [−0.087, −0.051]*	−0.053 [−0.075, −0.031]*
Novelty (child)	−0.012 [−0.022, −0.002]*	−0.002 [−0.021, 0.017]	0.001 [−0.023, 0.025]
Distance (parent to child)	0.040 [0.031, 0.049]*	0.028 [0.009, 0.046]*	0.044 [0.022, 0.066]*
Pop. size	0.075 [0.051, 0.098]*	0.040 [0.013, 0.067]*	0.149 [0.111, 0.186]*
Time	−0.437 [−0.446, −0.427]*	−0.430 [−0.447, −0.412]*	−0.293 [−0.315, −0.271]*

Table S4: The posterior means and 95% credible intervals for the grandchildren equation under a Poisson, a negative binomial, and the count part of a hurdle negative binomial. The negative binomial is the primary family for this outcome and its column is the one reproduced in Table 2, whereas the Poisson column is the fit that the comparison above rejects. Estimates whose intervals do not overlap zero are marked with an asterisk. Complexity reduces grandchildren under every family (−0.070, −0.069, −0.053), as do later submission times, whereas parent-child distance and population size raise them under every family, although the negative binomial estimates of the latter two are 30% and 47% smaller than those of the Poisson. Novelty is the exception: it is negative with an interval excluding zero under the Poisson only (−0.012 [−0.022, −0.002]), and its interval covers zero under both overdispersed families.

Outcome	Predictor	Est.	2.5%	97.5%	
# grandchildren	Complexity (child)	−0.053	−0.075	−0.031	*
# grandchildren	Novelty (child)	0.001	−0.023	0.025	
# grandchildren	Distance (parent to child)	0.044	0.022	0.066	*
# grandchildren	Pop. size	0.149	0.111	0.186	*
# grandchildren	Time	−0.293	−0.315	−0.271	*
# grandchildren (hurdle: P(0))	Complexity (child)	0.055	0.035	0.074	*
# grandchildren (hurdle: P(0))	Novelty (child)	0.027	0.006	0.047	*
# grandchildren (hurdle: P(0))	Distance (parent to child)	−0.021	−0.040	−0.001	*
# grandchildren (hurdle: P(0))	Pop. size	0.067	0.038	0.100	*
# grandchildren (hurdle: P(0))	Time	0.415	0.396	0.434	*

Table S5: Both parts of the hurdle negative binomial fit of the grandchildren equation. The first block is the count part, which applies to images that are remixed at least once and repeats the last column of Table S4. The second block is the hurdle part, in which a positive coefficient means that an image is more likely to receive no remixes at all. The two parts share the same five predictors and the same random intercepts for tool, party, and creator. Novelty only appears in the hurdle part (0.027 [0.006, 0.047] on the probability of a zero, against 0.001 [−0.023, 0.025] on the count), so more novel images are more likely to go unremixed but are not remixed fewer times when they are remixed at all. Complexity acts on both parts in the same direction (0.055 on the probability of a zero, −0.053 on the count). Population size splits between the two: larger parties produce more images that receive no remixes (0.067) and more remixes among those that are remixed (0.149), which is why the single coefficient from the negative binomial (0.040) is the smallest of the three families. Time behaves the same way, and runs mostly through the hurdle part (0.415 against −0.293). Parent-child distance is the one predictor that lowers the probability of a zero (−0.021).

Outcome	Predictor	Est.	2.5%	97.5%	
Complexity (child)	# parents	0.011	0.001	0.021	*
Complexity (child)	Complexity (parent image)	0.442	0.436	0.448	*
Complexity (child)	Novelty (parent image)	0.052	0.045	0.059	*

Outcome	Predictor	Est.	2.5%	97.5%	
Complexity (child)	Pop. size	−0.045	−0.061	−0.028	*
Complexity (child)	Time	−0.010	−0.016	−0.003	*
Complexity (child)	Depth	0.009	0.002	0.016	*
Complexity (child)	Multiple parents (indicator)	−0.021	−0.040	−0.001	*
Complexity (child)	Prompt exists (indicator)	0.021	0.004	0.039	*
Complexity (child)	Distance (b/t parent images)	−0.000	−0.008	0.008	
Complexity (child)	Length (parent text)	0.078	0.067	0.089	*
Complexity (child)	Novelty (parent text)	0.042	0.033	0.051	*
Complexity (child)	Distance (parent text to parent images)	0.006	−0.002	0.013	
Novelty (child)	# parents	−0.072	−0.082	−0.063	*
Novelty (child)	Complexity (parent image)	0.057	0.051	0.063	*
Novelty (child)	Novelty (parent image)	0.453	0.447	0.460	*
Novelty (child)	Pop. size	0.020	0.008	0.033	*
Novelty (child)	Time	−0.010	−0.017	−0.004	*
Novelty (child)	Depth	−0.003	−0.010	0.003	
Novelty (child)	Multiple parents (indicator)	−0.178	−0.197	−0.160	*
Novelty (child)	Prompt exists (indicator)	0.160	0.142	0.177	*
Novelty (child)	Distance (b/t parent images)	0.035	0.027	0.043	*
Novelty (child)	Length (parent text)	−0.013	−0.024	−0.003	*
Novelty (child)	Novelty (parent text)	0.251	0.243	0.259	*
Novelty (child)	Distance (parent text to parent images)	0.005	−0.002	0.013	
Distance (child)	# parents	0.180	0.172	0.189	*
Distance (child)	Complexity (parent image)	−0.008	−0.013	−0.003	*
Distance (child)	Novelty (parent image)	0.260	0.254	0.266	*
Distance (child)	Pop. size	0.054	0.041	0.068	*
Distance (child)	Time	−0.013	−0.019	−0.007	*
Distance (child)	Depth	−0.037	−0.043	−0.032	*
Distance (child)	Multiple parents (indicator)	0.049	0.033	0.066	*
Distance (child)	Prompt exists (indicator)	0.091	0.076	0.107	*
Distance (child)	Distance (b/t parent images)	0.347	0.339	0.354	*
Distance (child)	Length (parent text)	0.011	0.001	0.021	*
Distance (child)	Novelty (parent text)	−0.024	−0.031	−0.016	*
Distance (child)	Distance (parent text to parent images)	0.508	0.501	0.514	*
# likes	Complexity (child)	0.002	−0.005	0.009	
# likes	Novelty (child)	0.040	0.034	0.047	*
# likes	Distance (parent to child)	−0.011	−0.017	−0.005	*
# likes	Pop. size	−0.241	−0.281	−0.197	*
# likes	Time	−0.238	−0.245	−0.232	*
# grandchildren	Complexity (child)	−0.070	−0.079	−0.061	*
# grandchildren	Novelty (child)	−0.012	−0.022	−0.002	*
# grandchildren	Distance (parent to child)	0.041	0.031	0.050	*
# grandchildren	Pop. size	0.075	0.050	0.100	*
# grandchildren	Time	−0.437	−0.446	−0.427	*

Table S6: The estimates for all five equations when the multiple-parents and prompt-used indicators are included as main effects in the three child equations, in the same layout as Table 2. No coefficient changes sign relative to Table 2. Two small coefficients gain an interval excluding zero: the number of parents on complexity (0.011 [0.001, 0.021], against 0.005 [−0.003, 0.012]) and prompt length on parent-child distance (0.011 [0.001, 0.021], against −0.001 [−0.010, 0.009]). The number-of-parents coefficient on novelty shrinks from −0.130 to −0.072 and prompt length on novelty from −0.037 to −0.013, both still excluding zero, and the outcome equations are unchanged. Images with multiple parents are less novel

(−0.178), slightly less complex (−0.021), and further from their parents (0.049), whereas prompted images are more novel (0.160), slightly more complex (0.021), and further from their parents (0.091).

Outcome	Bayes R2	2.5%	97.5%
Complexity (child)	0.455	0.451	0.459
Novelty (child)	0.502	0.498	0.506
Distance (child)	0.581	0.578	0.584
# likes	0.428	0.422	0.434
# grandchildren	0.090	0.087	0.093

Table S7: The Bayes R^2 of the indicator model, for comparison with Table S3. The values are within 0.007 of those from the main model in every equation.

Outcome	Predictor	Est.	2.5%	97.5%	
Complexity (child)	# parents	0.002	−0.007	0.012	
Complexity (child)	Complexity (parent image)	0.579	0.570	0.588	*
Complexity (child)	Novelty (parent image)	0.051	0.040	0.062	*
Complexity (child)	Distance (b/t parent images)	−0.000	−0.010	0.008	
Complexity (child)	Pop. size	−0.044	−0.061	−0.026	*
Complexity (child)	Time	−0.012	−0.020	−0.004	*
Complexity (child)	Depth	0.016	0.007	0.024	*
Complexity (child)	Length (parent text)	0.068	0.053	0.083	*
Complexity (child)	Novelty (parent text)	0.025	0.013	0.036	*
Complexity (child)	Distance (parent text to parent images)	0.005	−0.008	0.019	
Novelty (child)	# parents	−0.081	−0.089	−0.072	*
Novelty (child)	Complexity (parent image)	0.068	0.060	0.077	*
Novelty (child)	Novelty (parent image)	0.647	0.637	0.658	*
Novelty (child)	Distance (b/t parent images)	−0.047	−0.056	−0.038	*
Novelty (child)	Pop. size	0.012	−0.003	0.027	
Novelty (child)	Time	−0.010	−0.018	−0.003	*
Novelty (child)	Depth	−0.001	−0.009	0.007	
Novelty (child)	Length (parent text)	−0.028	−0.042	−0.014	*
Novelty (child)	Novelty (parent text)	0.204	0.193	0.214	*
Novelty (child)	Distance (parent text to parent images)	0.057	0.045	0.070	*
Distance (child)	# parents	0.194	0.188	0.201	*
Distance (child)	Complexity (parent image)	0.001	−0.005	0.006	
Distance (child)	Novelty (parent image)	0.201	0.194	0.208	*
Distance (child)	Distance (b/t parent images)	0.411	0.405	0.417	*
Distance (child)	Pop. size	0.023	0.012	0.034	*
Distance (child)	Time	−0.006	−0.011	−0.001	*
Distance (child)	Depth	−0.015	−0.020	−0.010	*
Distance (child)	Length (parent text)	−0.005	−0.015	0.005	
Distance (child)	Novelty (parent text)	0.011	0.004	0.018	*
Distance (child)	Distance (parent text to parent images)	0.347	0.338	0.355	*

Table S8: A complete-case refit of the three child equations on multi-parent images only (n = 40,879), with the between-parent distance entered as a plain main effect and no imputation. This is the only subset in which between-parent distance is actually observed. Its coefficient on child novelty reverses sign, from 0.030 [0.022, 0.038] in Table 2 to −0.047 [−0.056, −0.038], whereas on child complexity it remains null and on parent-child distance it is larger (0.411 against 0.347). Within this subset, between-parent distance and mean parent novelty are correlated at r = −0.56 and the parent novelty slope is much steeper (0.647 against 0.457), so the partial effect of distance changes sign. The population size coefficient on

novelty also loses its interval here (0.012 [−0.003, 0.027]), and the prompt novelty coefficient on parent-child distance changes sign (0.011 against −0.024).

Outcome	Predictor	Est.	2.5%	97.5%	
Complexity (child)	# parents	0.015	0.005	0.024	*
Complexity (child)	Complexity (parent image)	0.399	0.392	0.407	*
Complexity (child)	Novelty (parent image)	0.055	0.047	0.063	*
Complexity (child)	Length (parent text)	0.077	0.065	0.089	*
Complexity (child)	Novelty (parent text)	0.044	0.035	0.053	*
Complexity (child)	Distance (parent text to parent images)	0.002	−0.006	0.010	
Complexity (child)	Pop. size	−0.048	−0.065	−0.030	*
Complexity (child)	Time	−0.006	−0.014	0.002	
Complexity (child)	Depth	0.007	−0.001	0.015	
Complexity (child)	Distance (b/t parent images)	0.003	−0.008	0.014	
Novelty (child)	# parents	−0.126	−0.135	−0.116	*
Novelty (child)	Complexity (parent image)	0.055	0.048	0.062	*
Novelty (child)	Novelty (parent image)	0.418	0.410	0.426	*
Novelty (child)	Length (parent text)	−0.009	−0.021	0.003	
Novelty (child)	Novelty (parent text)	0.265	0.256	0.273	*
Novelty (child)	Distance (parent text to parent images)	0.004	−0.003	0.013	
Novelty (child)	Pop. size	0.022	0.006	0.039	*
Novelty (child)	Time	−0.012	−0.021	−0.004	*
Novelty (child)	Depth	0.003	−0.005	0.011	
Novelty (child)	Distance (b/t parent images)	0.023	0.013	0.034	*
Distance (child)	# parents	0.163	0.153	0.172	*
Distance (child)	Complexity (parent image)	−0.007	−0.014	−0.000	*
Distance (child)	Novelty (parent image)	0.262	0.255	0.270	*
Distance (child)	Length (parent text)	0.003	−0.008	0.014	
Distance (child)	Novelty (parent text)	−0.025	−0.033	−0.017	*
Distance (child)	Distance (parent text to parent images)	0.521	0.514	0.529	*
Distance (child)	Pop. size	0.060	0.044	0.077	*
Distance (child)	Time	−0.013	−0.020	−0.005	*
Distance (child)	Depth	−0.044	−0.052	−0.037	*
Distance (child)	Distance (b/t parent images)	0.274	0.263	0.284	*

Table S9: A complete-case refit of the three child equations on prompted images only (n = 44,220), with the three prompt variables entered as plain main effects and no imputation (the between-parent distance keeps its indicator interaction). Mean imputation does not attenuate the prompt coefficients: prompt novelty predicts child novelty (0.265 against 0.253 in Table 2) and complexity (0.044 against 0.042), and prompt length predicts complexity (0.077 against 0.075), all of which are marginally larger than in the imputed fit. One coefficient does shrink, prompt length on child novelty, from −0.037 to −0.009 [−0.021, 0.003]. The small time and depth coefficients on complexity lose their intervals in this subset.
